# Design optimization of antibody-ligand motifs to enhance CAR-T redirection activity against solid tumors

**DOI:** 10.1016/j.xcrm.2026.102885

**Published:** 2026-06-25

**Authors:** Xuechun Wang, Shuhong Li, Qiaoru Guo, Licai Shi, Jian Guo, Xuexiu Qi, Xiaoyi Wei, Qingen Da, Fang Huang, Kunfu Ouyang, Yang Xu, Jun Li, Yu J. Cao

**Affiliations:** 1 State Key Laboratory of Chemical Oncogenomics, Shenzhen Key Laboratory of Chemical Genomics, Peking University Shenzhen Graduate School, Shenzhen, Guangdong 518055, China; 2 Department of Human Cell Biology and Genetics, School of Medicine, Southern University of Science and Technology, Shenzhen, Guangdong 518055, China; 3 Department of Cardiovascular Surgery, Peking University Shenzhen Hospital, Shenzhen, Guangdong 518036, China; 4 Suzhou Yoda Biotechnology, Inc, Suzhou, Jiangsu 215200, China; 5 Institute of Chemical Biology, Shenzhen Bay Laboratory, Shenzhen, Guangdong 518132, China

**Keywords:** switchable CAR-T, antibody, ligand, Her2, ROR1, VEGF, VEGFR, tumor microenvironment

## Abstract

Antigenic heterogeneity and the tumor microenvironment remain major obstacles to effective chimeric antigen receptor T (CAR-T) therapy, but natural ligands engaging multiple antigens within tumors and their milieu offer a promising solution. Here, we present a multitarget switchable CAR-T (sCAR-T) strategy that integrates a universal receptor on T cells with an antibody-ligand motif, combining an anti-Her2 single-chain variable fragment (scFv) (4D5) and spliced VEGF-A (VEGF121) to target Her2, VEGFR1, and VEGFR2. Optimization of the switch and CAR hinge preserved the ligand’s native dimeric conformation, enhancing antigen recognition and promoting immunological synapse formation. In syngeneic and xenograft models, sCAR-T achieves superior tumor eradication and vasculature disruption compared with conventional CAR-T, overcoming immune escape driven by antigenic heterogeneity. We further extend the sCAR-T design to target receptor tyrosine kinase-like orphan receptor 1 (ROR1), demonstrating that the antibody-ligand motif-based strategy provides a versatile framework for complex antigen targeting, with potential to improve efficacy and safety and enable broader application in immunotherapies targeting complex antigenic combinations.

## Highlights

• Switches and CAR hinge optimization enhance IS formation and efficacy
• sCAR-T yields potent antitumor efficacy and safety vs. conventional CAR-T *in vivo*
• Robust tumor vascular killing activity is observed in sCAR-T
• sCAR-T efficiently overcomes tumor immune evasion

## Graphical abstract

**Figure:**
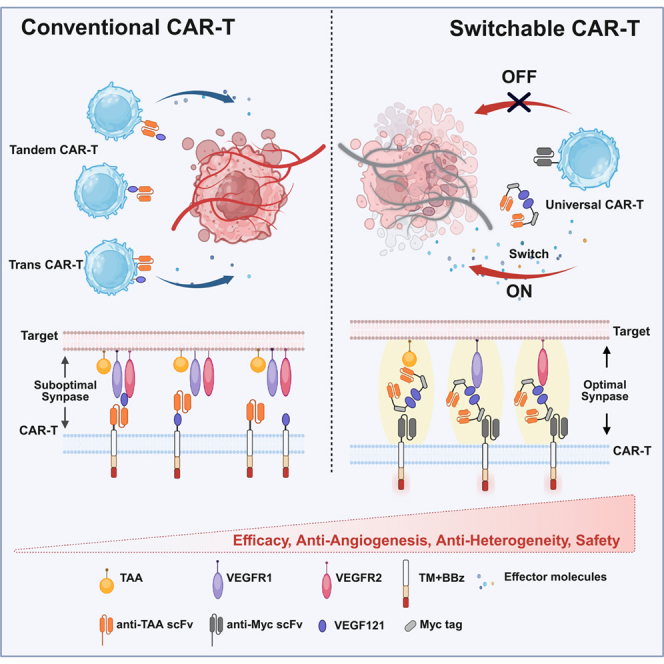

## Teaser

Wang et al. designed a sCAR-T to overcome solid tumor heterogeneity and the tumor microenvironment. The fusion of scFv with ligands enables coordinated targeting of multiple antigens. Optimization of switches and CAR hinges facilitates IS formation and exhibits potent antitumor efficacy and controllable safety in both syngeneic and xenogeneic *in vivo* models.

## Introduction

Chimeric antigen receptor T (CAR-T) cell therapy has achieved remarkable success in treating hematologic malignancies; however, its efficacy against solid tumors remains limited by multiple interconnected challenges. High antigenic heterogeneity in solid tumors is a major contributor to immune escape and treatment failure, while additional barriers include off-target toxicity, inefficient T cell trafficking to tumor sites, and a highly suppressive tumor microenvironment that impairs CAR-T cell infiltration, activation, and persistence. Tumor heterogeneity not only suppresses CAR-T cell recognition of tumor cells but also facilitates immune evasion through diverse mechanisms.^1^ To address this issue, CAR-T cells with the ability to recognize and bind multiple antigens simultaneously, such as Her2/MUC2, PSCA/MUC1, and B7-H3/GD2,^2^^,^^3^^,^^4^ have been developed, with the aim of reducing immune escape and enhancing tumor clearance. Despite these advances, their effectiveness is constrained by inherent limitations of single-chain variable fragments (scFvs),^5^^,^^6^ which, while widely utilized in CAR-T cell designs for their modularity and specificity, are prone to structural instability under variable temperature and pH conditions^7^ and to mismatches between light and heavy chains.^8^^,^^9^^,^^10^ Therefore, addressing these vulnerabilities is crucial for the development of robust CAR-T cell therapies that can effectively target the complex and dynamic landscape of solid tumors.

Natural ligands are widely used in biomedicine, including delivery, diagnosis, imaging, and cancer therapy.^11^^,^^12^^,^^13^^,^^14^^,^^15^ When incorporated into CAR-T cell designs, these ligands offer unique advantages, such as simplified domain recognition and the ability to target multiple receptors, namely, achieving multitargeting functionality through a single recognition structure. For example, preclinical CAR-T cells constructed with growth arrest-specific protein 6 (GAS6), a ligand of AXL, MERTK, and TYRO3, have exhibited impressive killing efficacy not only against tumor cells but also against tumor stem cell-like cells, which are often resistant to conventional therapies.^16^ Similarly, ligands such as vascular endothelial growth factor C (VEGF-C) have been integrated into CAR-T cell constructs to target VEGFR2 and VEGFR3, enabling tumor vasculature disruption and prolonging treatment duration.^17^ A key feature of most natural ligands is their existence as dimers, trimers, or higher order oligomers, a structural characteristic often emulated in conventional CAR-T cell designs to optimize their functional mimicry. However, accurately replicating these multimeric configurations has proven technically challenging, often compromising the normal recognition and binding functions of the ligands,^18^ leading to reduced efficacy and stability.^19^

Switchable CAR-T (sCAR-T) cells represent an innovative approach to CAR design. Unlike conventional CARs, they employ a split architecture composed of two components: an antigen-targeting moiety, termed the “switch,” and a universal receptor expressed on T cells.^20^^,^^21^^,^^22^^,^^23^^,^^24^^,^^25^^,^^26^^,^^27^^,^^28^ This modular architecture allows the antigen-recognition switch to function independently of T cells, offering increased flexibility through the incorporation of various linkers and arrangements to optimize the recognition structure. Leveraging this strategy, our group previously developed APRIL/BAFF sCAR-T cells with broader B cell malignancy targeting than conventional CAR-T cells.^29^ For solid tumors, we pioneered the design of bispecific sCAR-T cells targeting Her2 and IGF1R receptors, which resulted in increased cytotoxic activity against breast cancer with low Her2 expression.^30^ Nevertheless, the *in vivo* data failed to meet expectations, highlighting the complexity of translating these designs into effective therapies for solid tumors. These findings underscore the critical need for structural optimization when designing sCAR-T cells with different antibody-ligand motifs; striking a balance between synergistic functionality and minimizing off-target toxicity remains a key challenge.^30^

In this study, we present an antibody-ligand-based sCAR-T platform combining 4D5 scFv and VEGF121 for synergistic activity against Her2 and VEGFR1/2 in solid tumors and tumor vasculature. By optimizing the switch structure and hinge region, we successfully promoted the formation of immunological synapses (ISs) between effector and target cells, thereby promoting robust inflammatory cytokine release. In syngeneic and xenograft models, sCAR-T disrupted tumor vasculature, strengthening antitumor efficacy. Furthermore, in a mixed tumor model reflecting the antigenic heterogeneity commonly observed in solid tumors, sCAR-T cells showed a remarkable capacity to counteract tumor immune escape, highlighting their superior efficacy compared with conventional CAR-T cell therapy and single-target CAR-T cell combinations. Importantly, we further extended this modular antibody-ligand motif-based strategy to target receptor tyrosine kinase-like orphan receptor 1 (ROR1), demonstrating the platform’s adaptability and potential applicability across diverse tumor antigens. In conclusion, this modular CAR-T cell strategy demonstrates a proof of concept for enhancing antigen recognition and functional adaptability and may provide a promising avenue to overcome antigenic heterogeneity and tumor microenvironmental barriers in solid tumors.

## Results

### Design and characterization of the antibody-ligand-based conventional CAR-T cells

**Figure 1:**
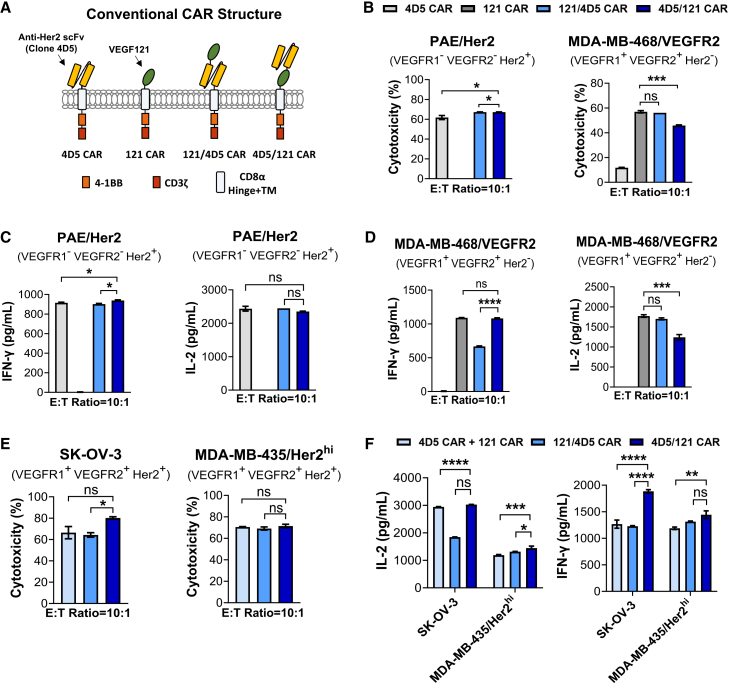
Antitumor efficacy of antibody-ligand-based conventional tandem CAR-T cells *in vitro* (A) Schematic of antibody-ligand-based conventional tandem CAR structures. (B) Cytotoxicity assay of conventional CAR-T cells toward single-positive PAE (Her2^+^) or MDA-MB-468 (VEGFR1^+^ VEGFR2^+^) cells. The 4D5 CAR, 121 CAR, 4D5/121 CAR, and 121/4D5 CAR-T cells were coincubated with the targeted cells at an E:T ratio of 10:1 for 24 h (*n* = 3). Cytolytic activity was evaluated using a lactate dehydrogenase (LDH) release assay. ∗*p* < 0.05 and ∗∗∗*p* < 0.001 by one-way ANOVA, means ± SD. (C) Inflammatory cytokine (human IL-2 and human IFN-γ) release levels. CAR-T cells were coincubated with target cells at an E:T ratio of 10:1, and the supernatant was collected for ELISA (*n* = 3). ∗*p* < 0.05 by one-way ANOVA, means ± SD. (D) Inflammatory cytokine (human IL-2 and human IFN-γ) release levels. CAR-T cells were coincubated with target cells at an E:T ratio of 10:1, and the supernatant was collected for ELISA (*n* = 3). ∗∗∗*p* < 0.001 and ∗∗∗∗*p* < 0.0001 by one-way ANOVA, means ± SD. (E) Cytotoxicity assay of conventional CAR-T cells to SK-OV-3 (VEGFR^+^ VEGFR2^+^ Her2^+^) and MDA-MB-435/Her2^hi^ (VEGFR^+^ VEGFR2^+^ Her2^+^) cell lines. 4D5/121 CAR, 121/4D5 CAR, and the combination of 4D5 CAR and 121 CAR-T cells were coincubated with SK-OV-3 and MDA-MB-435/Her2^hi^ cells at an E:T ratio of 10:1 for 24 h (*n* = 3). Cytolytic activity was evaluated using an LDH release assay. ∗*p* < 0.05 by one-way ANOVA, means ± SD. (F) Inflammatory cytokine (human IL-2 and human IFN-γ) release levels. CAR-T cells were coincubated with target cells at an E:T ratio of 10:1 for 24 h, and the supernatant was collected for ELISA (*n* = 3). ∗*p* < 0.05, ∗∗*p* < 0.01, ∗∗∗*p* < 0.001, and ∗∗∗∗*p* < 0.0001 by two-way ANOVA, means ± SD. See also Figures S1.

Given the untapped potential of natural ligands in CAR-T cell therapy, we utilized an antibody-ligand combination strategy to generate a conventional tandem CAR construct. The anti-Her2 scFv (clone 4D5, designated “4D5”) from trastuzumab and VEGF121 (the natural ligand for VEGFR1 and VEGFR2, designated “121”)^31^^,^^32^^,^^33^^,^^34^ were introduced to form the antigen-binding domain of a second-generation CAR containing a 4-1BB costimulatory domain,^35^^,^^36^ and the CAR expression levels were confirmed by flow cytometry analysis (Figures 1A and S1A). Cytotoxicity assays were performed on Her2- or VEGFR1/2-positive cell lines to evaluate tandem CAR-T activity. For the Her2-positive cell line, the two conventional tandem CAR-T cells exhibited comparable cytotoxicity (Figure 1B) and proinflammatory cytokine secretion profiles (Figures 1C, 1D, and S1B) to those of single-target CAR-T cells. However, when VEGF121 was positioned at the proximal membrane terminus of the tandem CAR construct (4D5/121 CAR), the ability of CAR-T cells to kill Her2-negative cells was markedly decreased. Moreover, compared with single-target CAR-T cells, conventional tandem CAR-T cells demonstrated similar cytotoxicity and greater proinflammatory cytokine secretion in Her2/VEGFR1/VEGFR2 triple-positive cell lines, but this effect was observed only in the 4D5/121 CAR-T cells and was not evident in the 121/4D5 CAR-T cells (Figures 1E, 1F, and S1C). Overall, we concluded that the combination of the 4D5 scFv and the ligand VEGF121 within the conventional tandem CAR framework did not significantly improve the ability of CAR-T cells to target tumor cells. This observation appeared to be related to the positioning of the 4D5 scFv and VEGF121. Furthermore, we hypothesized that the cell membrane anchoring restricted VEGF121 dimerization and 4D5 recognition, reducing binding and antitumor activity. Therefore, the multitarget CAR-T cell strategy employing an antibody-ligand combination still required further optimization.

### Structural determination of antibody-ligand motifs

**Figure 2:**
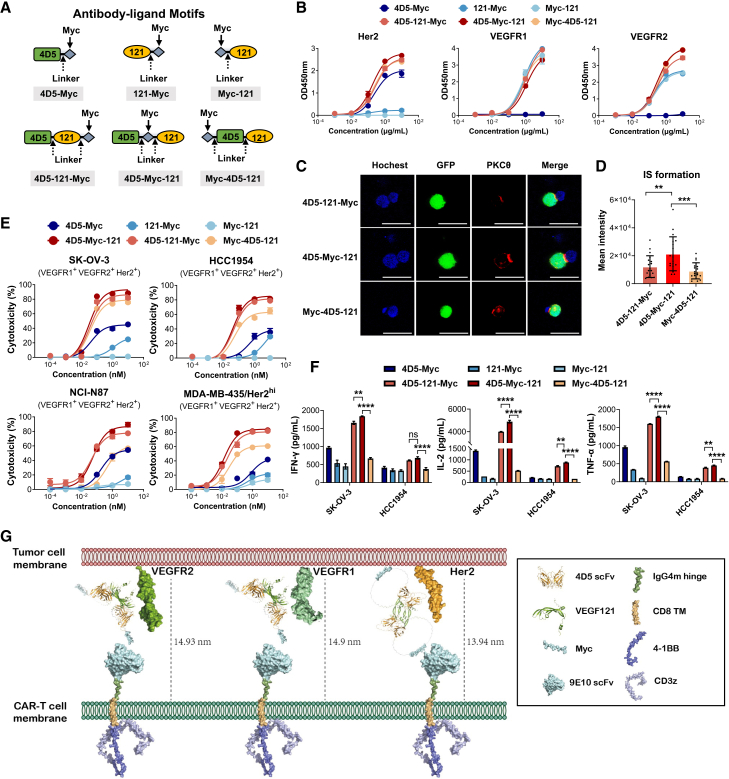
Achieving optimal antitumor efficacy by optimizing the antibody-ligand motif switch structure (A) Structure scheme of antibody-ligand motif switches. (B) Binding profiles of antibody-ligand motif switches to the antigens Her2, VEGFR1, and VEGFR2. (C) Confocal immunofluorescent of IS formation triggered by 4D5-121-Myc, 4D5-Myc-121, and Myc-4D5-121 (100 nM) in SK-OV-3 and 9E10-IgG4m CAR-T cells after 1 h of coincubation. PKC-θ was labeled to show IS formation. GFP (tumor cells, green), anti-PKC-θ (red), and Hoechst (nucleus, blue) were used. Scale bars, 10 μm. (D) Quantitative comparison of the cell contact area on the basis of the area of PKC-θ accumulation in the IS (*n* = 15). ∗∗*p* < 0.01 and ∗∗∗*p* < 0.001 by one-way ANOVA, means ± SD. (E) A cytotoxicity assay of different switches (E:T ratio of 10:1 with 10^−4^–10^1^ nM) was used for 24 h (*n* = 3). Cytolytic activity was evaluated via an LDH release assay. (F) ELISA quantification of human IL-2, IFN-γ, and TNF-α (E:T = 10:1, *n* = 3). ∗∗*p* < 0.01 and ∗∗∗∗*p* < 0.0001 by two-way ANOVA, means ± SD. (G) Scheme of the simulation structures of 4D5-Myc-121 interacting between Her2, VEGFR1, and VEGFR2 on the surface of tumor cell membrane and 9E10-IgG4m CAR on T cells. 4D5-Myc-121 and 9E10-IgG4m CAR structure predictions via the AlphaFold3 server. On the basis of the reported binding structures of 4D5 Fab with the Her2 receptor (PDB: 1n8z), VEGFA with VEGFR1 (PDB: 5t89), and VEGFA with VEGFR2 (PDB: 3v2a), a schematic of the 4D5-Myc-121 interactions with Her2, VEGFR1, and VEGFR2 was generated. See also Figures S2, S3, and S4; Tables S1 and S2.

To increase the effectiveness of antibody-ligand combinations in CAR-T cell therapy, we introduced a split-design approach in which CAR-T cells are divided into two components: an antigen-targeting moiety, referred to as the “switch,” and a universal receptor expressed on T cells that recognizes this switch to mediate cytotoxic functions. By fusing the targeting moieties to a short 10-amino acid (aa) peptide neoepitope (EQKLISEEDL) derived from the Myc tag, we constructed three switches with different arrangements (4D5-121-Myc, 4D5-Myc-121, and Myc-4D5-121) and control switches (4D5-Myc, 121-Myc, and Myc-121) (Figure 2A). By purifying the switch proteins via size-exclusion chromatography (SEC) and subsequently validating all the VEGF121-containing switches using primary mass spectrometry (Table S1), SDS-PAGE, and western blot analysis (Figures S2A and S2B), we confirmed that these VEGF121-based switches exhibit native homodimer structures (Table S2; Figure S2C). In addition, all switches showed similar Her2, VEGFR1, and VEGFR2 binding. These results show that homodimeric antibody-ligand motifs did not reduce receptor affinity compared to control switches with only an antibody or ligand added (Figure 2B).

For comparison, we used CAR-T cells with an anti-Myc scFv (clone 9E10) harboring an IgG4m hinge (9E10-IgG4m) as previously reported.^20^ The IS is a critical structure that enables T cells to bind to target cells and perform their immune functions.^37^ Protein kinase C theta (PKC-θ) plays a key role in regulating signaling within the IS.^38^^,^^39^^,^^40^ Measuring the PKC-θ signal intensity in the IS is essential for screening candidate structures with optimal activity. As shown in Figure 2C, the 4D5-Myc-121 switch promoted more efficient IS formation, and fluorescence quantification confirmed its ability to increase the fluorescence intensity of PKC-θ (Figure 2D). Additionally, the significant differences in the PKC-θ fluorescence intensity suggest that an optimal IS geometry is required for effective synapse formation. Intracellular calcium flux assays revealed rapid, robust calcium signaling in CAR-T cells. Notably, 4D5-Myc-121 induced the fastest onset and highest peak intensity, confirming that high-density ISs efficiently drive CAR-T activation (Figure S2D). 4D5-Myc-121 showed higher cytotoxicity than 4D5-121-Myc and Myc-4D5-121 (Figures 2E and S3A). Furthermore, coincubation with 4D5-Myc-121 promoted a significant increase in the release of proinflammatory cytokines (Figures 2F and S3B). Nevertheless, fusion of the Myc tag to the N terminus of 4D5 scFv (Myc-4D5-121) significantly reduced the cytotoxicity and cytokine release levels. This reduction may be attributed to the constrained antigen-binding domain of this construct, thereby impairing the redirection of CAR-T cells to tumor cells (Figures 2F and S3B).

CAR-antigen spatial distance is critical for T cell signaling initiation. The proximity between the two depends on various structural elements, including the location of the epitopes on the target molecule and the length of the linker connecting the scFv to the T cell membrane. Distances exceeding 15 nm allow CD45 and CD148 to enter the IS and block CAR-induced phosphorylation.^41^^,^^42^^,^^43^^,^^44^ To this end, we utilized the AlphaFold3 server to predict switch structures and compare antibody-ligand designs^45^ (Figures S4A–S4C). In addition, we determined the distance between the 4D5-Myc-121-mediated 9E10 scFv on the surface of CAR-T cells and Her2, VEGFR1, and VEGFR2 on the surface of tumor cells. We found that the distances between the 4D5-Myc-121-mediated CAR-T cells and the three antigens were consistently approximately 15 nm. These data strongly support 4D5-Myc-121 as the optimal switch structure (Figure 2G), and it was selected for further investigation in subsequent studies.

### Optimization of the sCAR-T cell design

**Figure 3:**
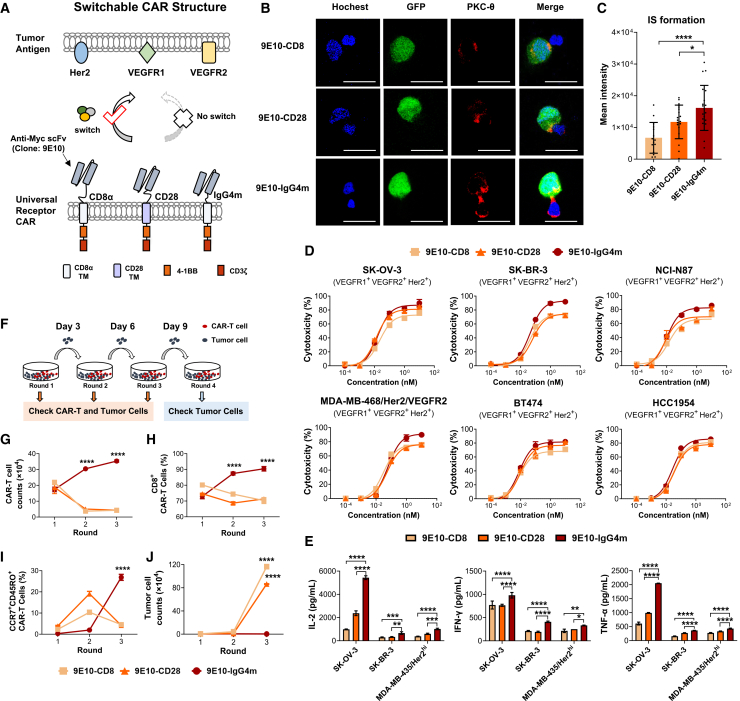
Achieving optimal antitumor efficacy by optimizing the hinge region of universal receptor CAR-T cells (A) Schematic of universal CARs with different hinge regions. (B) Confocal immunofluorescence of IS formation triggered by 4D5-Myc-121 (100 nM) in SK-OV-3 cells and 9E10-CD8, 9E10-CD28, or 9E10-IgG4m CAR-T cells after 1 h of coincubation. PKC-θ was labeled to show IS formation. GFP (tumor cells, green), anti-PKC-θ (red), and Hoechst (nucleus, blue) were used. Scale bars, 10 μm. (C) Quantitative comparison of the cell contact area on the basis of the area of PKC-θ accumulation in the IS (*n* = 15). ∗*p* < 0.05 and ∗∗∗∗*p* < 0.0001 by one-way ANOVA, means ± SD. (D) Cytotoxicity of sCAR-T cells against multiple tumor cell lines. 9E10-CD8, 9E10-CD28, and 9E10-IgG4m CAR-T cells were coincubated with target cells at an E:T ratio of 10:1, and 4D5-Myc-121 (10^−4^–10^1^ nM) was added for 24 h (*n* = 3). Cytolytic activity was evaluated via an LDH release assay. (E) ELISA quantification of human IL-2, IFN-γ, and TNF-α (E:T = 10:1, *n* = 3). ∗*p* < 0.05, ∗∗*p* < 0.01, ∗∗∗*p* < 0.001, and ∗∗∗∗*p* < 0.0001 by two-way ANOVA, means ± SD. (F) Schematic of the rechallenge assay *in vitro*. (G) Quantification of CD3^+^ CAR-T cells. Significance is marked as 9E10-IgG4m vs. 9E10-CD28. ∗∗∗∗*p* < 0.0001 by one-way ANOVA, means ± SD. (H) The proportion of CD8^+^ of CAR-T cells. Significance is marked as 9E10-IgG4m vs. 9E10-CD28. ∗∗∗∗*p* < 0.0001 by one-way ANOVA, means ± SD. (I) The ratio of central memory subtype of CAR-T cells (CCR7^+^/CD45RO^+^). Significance is marked as 9E10-IgG4m vs. 9E10-CD28. ∗∗∗∗*p* < 0.0001 by one-way ANOVA, means ± SD. (J) The number of GFP^+^ tumor cells. Significance is marked as 9E10-CD28 vs. 9E10-IgG4m and 9E10-CD8 vs. 9E10-IgG4m. ∗∗∗∗*p* < 0.0001 by one-way ANOVA, means ± SD. See also Figure S5.

The hinge regions of the CAR structure have been reported to regulate the proliferation and migration of CAR-T cells.^46^ Therefore, we introduced several hinge regions of varying lengths into CAR-T cells that target the Myc tag, including the CD8α hinge, CD28 hinge, and IgG4m hinge (derived from a dimeric mutant [S228P] of the IgG4 hinge)^23^ (Figure 3A). All three CARs showed comparable expression (Figure S5A). As shown in Figures 3B and 3C, the IgG4m hinge promoted more robust IS formation than other hinges. In intracellular calcium flux assays, 9E10-IgG4m CAR-T cells stimulated with 4D5-Myc-121 and tumor cells displayed the shortest onset time and highest peak intensity relative to other hinge variants (Figure S5B), indicating efficient early signaling. Moreover, the IgG4m hinge enhanced cytotoxicity (Figure 3D) and cytokine secretion (Figure 3E), supporting superior antitumor activity. Furthermore, multiple rounds of coculture assays (Figure 3F) to assess hinge effects on the long-term effector function of sCAR-T cells showed that repeated tumor cell stimulation enabled 9E10-IgG4m CAR-T cells to exhibit superior expansion (Figure 3G) and stable CD8^+^ T cell proportions (Figure 3H). Moreover, a decrease in PD-1 expression was observed after the first and second rounds of coculture (Figure S5C). Notably, the proportion of central memory cells among the 9E10-IgG4m CAR-T cells was greater after the third round (Figure 3I), indicating that compared to the other two hinge regions, the IgG4m hinge region reduced T cell exhaustion and improved T cell persistence. The complete elimination of tumor cells by 9E10-IgG4m CAR-T cells after three rounds of coculture (Figure 3I) further underscores the importance of maintaining T cell persistence. Tonic signaling, the spontaneous activation of signaling pathways under resting conditions, is a key determinant of CAR-T persistence.^47^^,^^48^ Assessment of Akt and ERK phosphorylation under antigen-free conditions revealed that IgG4m-hinged CAR-T cells exhibited lower tonic signaling compared with CD8- and CD28-hinged constructs (Figure S5D), thereby reducing premature activation, preventing exhaustion, and prolonging effector function. These findings provide evidence that the hinge region plays a crucial role in the proliferation and persistence of CAR-T cells and indicate that 9E10-IgG4m CAR-T cells facilitate antibody-ligand motif-mediated interactions between target cells and effector cells, thereby enhancing antigen-specific CAR-T cell activities.

### *In vitro* comparison of sCAR-T cells and conventional CAR-T cells

**Figure 4:**
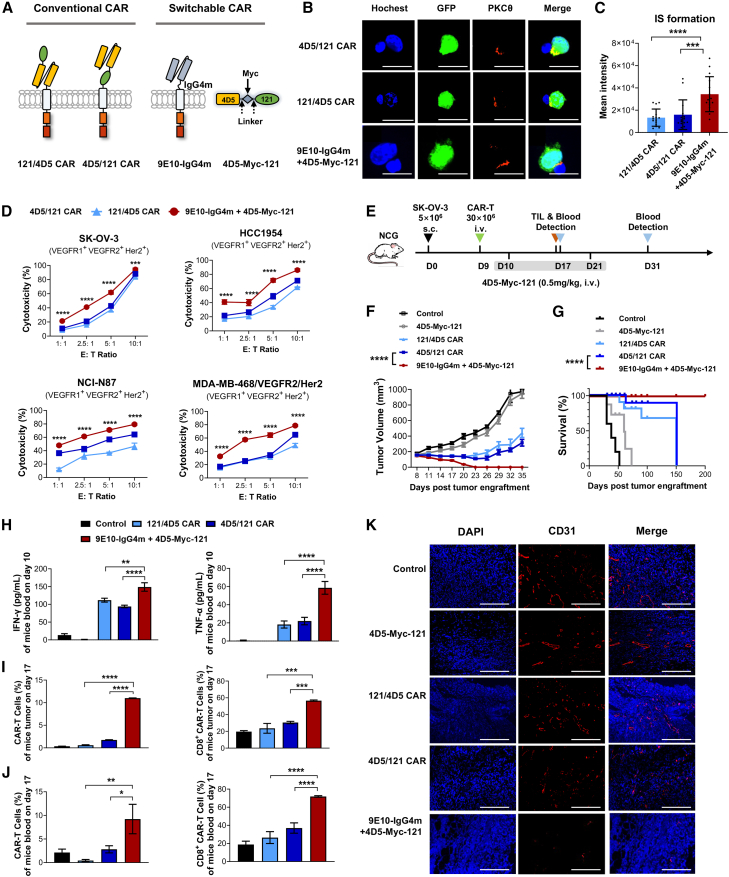
The antitumor efficacy of sCAR-T cells is superior to that of conventional CAR-T cells, both *in vitro* and *in vivo* (A) Schematic of conventional CAR and sCAR structures. (B) Confocal immunofluorescence of IS formation between 9E10-IgG4m triggered by 4D5-Myc-121 (100 nM) or 4D5/121 or 121/4D5 CAR-T cells and SK-OV-3 cells after 1 h of coincubation. PKC-θ was labeled to show IS formation. GFP (tumor cells, green), anti-PKC-θ (red), and Hoechst (nucleus, blue) were used. Scale bars, 10 μm. (C) Quantitative comparison of the cell contact area on the basis of the area of PKC-θ accumulation in the IS (*n* = 15). ∗∗∗*p* < 0.001 and ∗∗∗∗*p* < 0.0001 by one-way ANOVA, means ± SD. (D) Cytotoxicity of CAR-T cells to multiple tumor cell lines. The tumor cells were coincubated with 4D5/121 CAR, 121/4D5 CAR, and 9E10-IgG4m CAR-T cells supplemented with 1 nM 4D5-Myc-121 (E:T ratio = 1:1, 2.5:1, 5:1, 10:1; *n* = 3) for 24 h. Cytolytic activity was evaluated via an LDH release assay. ∗∗∗*p*＜0.001 and ∗∗∗∗*p*＜0.0001 by two-way ANOVA, means ± SD. (E) Schematic of the SK-OV-3 subcutaneous model in NCG mice. (F) Tumor volume (mm^3^ = length × width × height) (*n* = 5 or 7). ∗∗∗∗*p* < 0.0001 by two-way ANOVA, means ± SEM. (G) Kaplan-Meier survival curve of the mice (a tumor volume ≥2,000 was considered the terminal endpoint, *n* = 5). Significance is marked as 4D5/121 CAR vs. 9E10-IgG4m + 4D5-Myc-121 by the log rank (Mantel-Cox) test. ∗∗∗∗*p*＜0.0001. (H) Quantification of human IFN-γ and TNF-α levels in mouse blood serum on day 10 (*n* = 7). ∗∗*p* < 0.01 and ∗∗∗∗*p* < 0.0001 by two-way ANOVA, means ± SEM. (I) Tumor-infiltrating CAR-T cells (left) and CD8^+^ CAR-T cells (*n* = 3) (right) on day 17. ∗∗∗*p* < 0.001 and ∗∗∗∗*p* < 0.0001 by one-way ANOVA, means ± SEM. (J) Circulating CAR-T cells in mouse blood on day 17 (*n* = 7) and CD8^+^ of CAR-T cells in mouse blood on day 7. ∗*p* < 0.05, ∗∗*p* < 0.01, and ∗∗∗∗*p* < 0.0001 by one-way ANOVA, means ± SEM. (K) Immunofluorescence of CD31^+^ tumor blood vessels (*n* = 2), CD31 (red), and DAPI (blue). Scale bars, 100 μm. See also Figures S6, S7, S8, S9, S10, S11, and S12.

To determine whether the split design successfully preserved the respective activities of the antibodies and ligands, we compared conventional tandem CAR-T cells and sCAR-T cells (Figure 4A). Compared with conventional CAR-T cells, 9E10-IgG4m CAR-T cells triggered by 4D5-Myc-121 exhibited more pronounced IS formation and Ca^2+^ signaling (Figures 4B, 4C, and S6A), suggesting that the sCAR-T cells effectively maintained the optimal performance of the antibody-ligand combination strategy. sCAR-T induced higher target lysis at multiple E:T ratios (Figure 4D) and elevated proinflammatory cytokine secretion (Figure S6B). In multiple rounds of coculture with tumor cells, sCAR-T cells exhibited comparable proliferation to that of conventional tandem CAR-T cells (Figure S6C, left), with a significantly greater proportion of CD8^+^ cytotoxic T cells in the third round of coculture (Figure S6C, right). Moreover, a decrease in PD-1 expression was observed after the first and second rounds (Figure S6D). Until the third round of coculture, all the CAR-T cells exhibited similar antitumor abilities. Thus, we attempted a fourth round of coculture and surprisingly found that only the sCAR-T cells maintained sustained antitumor activity, whereas the conventional CAR-T cells became dysfunctional (Figure S6E). CD3ζ phosphorylation marks CAR-T activation; its reduction indicates functional impairment.^49^ Compared with conventional CAR-T cells, sCAR-T cells stimulated by tumor cells presented a greater level of CAR-CD3ζ phosphorylation (Figure S7A). This finding indicated that sCAR-T cells received more antigenic stimulation signals within the same period. Together, our findings highlight the essential role of the split-design CAR approach in preserving the original binding activity of the antibody/ligand while maintaining robust antitumor activity.

### The antibody-ligand motif enhances the efficacy of sCAR-T cells against solid tumors *in vivo*

To validate the *in vivo* efficacy of the split-design-based antibody-ligand combination strategy, we injected SK-OV-3 cells into immunodeficient mice to establish a human ovarian tumor model. The experimental timeline is shown in Figure 4E. Mice treated with sCAR-T cells showed complete tumor regression without recurrence and significantly prolonged overall survival (Figures 4F and 4G). Importantly, no treatment-related significant body weight loss or unexpected non-tumor-related deaths were detected (Figure S7B). Serum proinflammatory cytokines were significantly elevated on day 10 (Figures 4H and S7C), with IL-2 levels remaining high even on day 17 (Figure S7C). Further analysis of tumor samples on day 17 showed increased sCAR-T cell infiltration into the tumor microenvironment compared with conventional CAR-T cells (Figures 4I, left; and S7D). Consistent with the *in vitro* results (Figure S6C), the proportion of CD8^+^ sCAR-T cells in tumors was also higher (Figure 4I, right). These tumor-infiltrating CAR-T cells exhibited a pronounced effector memory phenotype (Figure S8A). Tumor recurrence occurred in both conventional CAR-T groups: day 23 for 121/4D5 CAR-T and day 26 for 4D5/121 CAR-T. In contrast, tumors in the sCAR-T cell group were completely eliminated by day 35. We then analyzed circulating CAR-T cells in peripheral blood. As expected, the proportions of CAR-T cells and CD8^+^ CAR-T cells were highest in the sCAR-T group on day 17 (Figure 4J). However, by day 31, the proportion of CD8^+^ T cells in the blood (Figure S8B, left) remained greater in the sCAR-T cell group than in the other groups, although there was no significant difference in the CAR-T cell content among the groups (Figure S8B, right), highlighting the persistence and therapeutic duration of sCAR-T cells. Safety assessment showed no obvious lung, liver, spleen, or heart toxicity in any CAR-T treatment group (Figure S8C). To further investigate the structural advantages of the sCAR-T design, we generated trans-4D5/121 CAR-T cells as an additional control (Figures S9A and S9B). *In vitro*, sCAR-T cells exhibited significantly higher target cell lysis across multiple E:T ratios (Figure S9C) and increased proinflammatory cytokine release (Figure S9D). Under the experimental framework in Figure S10A, conventional tandem CAR-T cells (4D5/121 CAR and 121/4D5 CAR), transCAR-T cells (trans-4D5/121 CAR), and sCAR-T cells were adoptively transferred into SK-OV-3 tumor-bearing mice. sCAR-T treatment led to complete tumor eradication by day 25, whereas trans-4D5/121 CAR-T cells, despite initial activity, allowed tumor recurrence starting on day 31 (Figure S10B). No treatment-related body weight loss or non-tumor-related deaths were observed (Figure S10C), and proinflammatory cytokines were evaluated on day 10 (Figure S10D). Tumor analyses on day 17 and day 24 revealed superior infiltration of sCAR-T cells into the tumor microenvironment compared with conventional tandem and transCAR-T cells (Figures S11A, left; S11B, left; and S11C), and the proportion of CD8^+^ sCAR-T cells remained elevated through day 24 (Figures S11A, right; and S11B, right). Peripheral blood analysis 4 weeks post-treatment (Figure S11D) showed stable CAR-T frequencies, with significantly higher circulating CD8^+^ CAR-T cells in the sCAR-T group. High levels of circulating CD8^+^ T cells were maintained even 10 days after drug withdrawal (Figure S11E). Notably, one mouse in the trans-4D5/121 CAR-T group developed severe ascites on day 38, indicating potential toxicity of the transCAR configuration (data not shown). Collectively, these results demonstrate that the antibody-ligand combination in sCAR-T cells enhances both antitumor efficacy and therapeutic persistence in solid tumors without inducing organ toxicity, substantially improving the overall therapeutic index.

### sCAR-T cells alleviate vascular abnormalities in the tumor microenvironment

Abnormal tumor blood vessels provide tumor cells with nutrients and prevent lymphocyte infiltration.^50^^,^^51^ VEGFR1 and VEGFR2 are key signaling factors of physiological angiogenesis and major therapeutic targets.^52^^,^^53^ However, although sCAR-T cells did not demonstrate a clear advantage in the disruption of HUVEC tube formation^54^ *in vitro*, which may be attributed to their short duration of action (Figures S12A and S12B), these results primarily reflect the capacity of CAR-T cells to disrupt vascular structures in the absence of tumor cell stimulation. CD31^+^ staining^55^ revealed that VEGF121-based CAR-T cells alleviated tumor vascular abnormalities. Notably, 9E10-IgG4m CAR-T cells treated with 4D5-Myc-121 almost completely abrogated the vascular abnormalities (Figures 4K and S12C). These results suggest that sCAR-T cells constructed with VEGF121 can remodel the tumor microenvironment by abrogating vascular abnormalities. Therefore, sCAR-T cells in combination with antitumor and antiangiogenic therapeutic strategies have great therapeutic potential in cancer.

### Murine sCAR-T cells enhance antitumor efficacy in an immunocompetent model

To further validate the clinical potential of the antibody-ligand sCAR-T cell strategy, we generated murine CAR-T cells to evaluate their antitumor potential in an immunocompetent environment. We constructed two tandem CAR-Ts (m4D5/121 and m121/4D5) and three anti-Myc CAR-Ts (m9E10) with different hinges (CD8α, CD28, and IgG4m), analogous to human CAR designs (Figures 1A and 3A). For optimal results, we constructed third-generation CAR constructs using retroviral vectors and performed subsequent validation, and murine CAR expression was detected by flow cytometry analysis (Figure S13A). The CT26 and 4T1^32^^,^^53^^,^^56^^,^^57^^,^^58^ cell lines, which express murine VEGFR1 and VEGFR2, were selected and engineered to overexpress human Her2, resulting in the construction of the CT26/Her2 and 4T1/Her2 cell lines. Compared with single-target CAR-T cells, conventional tandem CAR-T cells exhibited greater cytotoxic activity and significantly increased secretion of proinflammatory cytokines (Figures S13B and S13C). With respect to the split design of the antibody-ligand CAR-T cells, which was supported by the cytotoxicity and cytokine level data, we confirmed that the 4D5-Myc-121 switch resulted in increased antitumor activity (Figures S14A and S14B). In addition, consistent with the findings in the human sCAR-T cell experiments, the IgG4m hinge region significantly enhanced the cytotoxicity of sCAR-T cells and increased cytokine secretion (Figures S14C and S14D).

**Figure 5:**
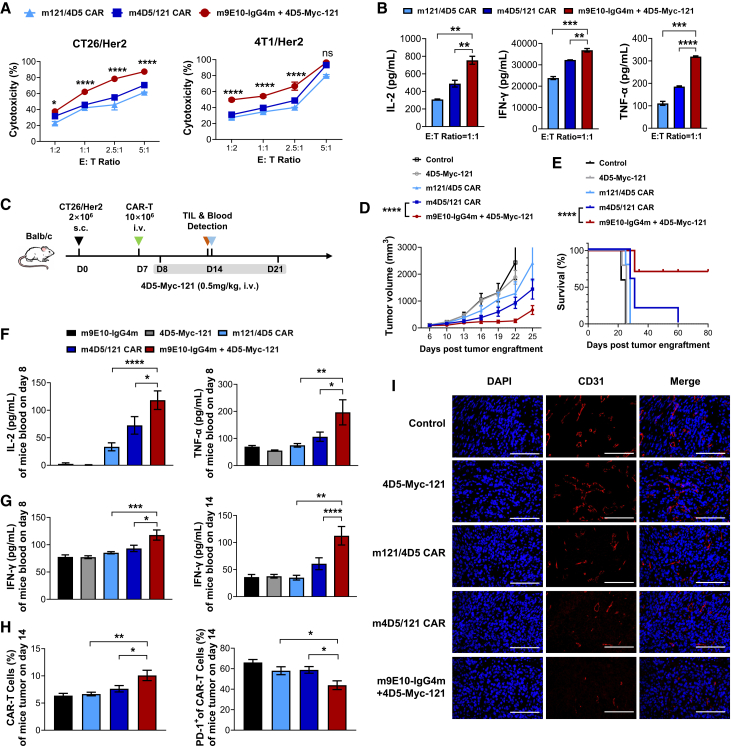
The antitumor efficacy of murine sCAR-T cells is superior to that of conventional murine CAR-T cells both *in vitro* and *in vivo* (A) CT26/Her2 and 4T1/Her2 cell lines were coincubated with m4D5/121 CAR, m121/4D5 CAR, or m9E10-IgG4m CAR-T cells supplemented with 1 nM 4D5-Myc-121 for 24 h (E:T ratio = 1:2, 1:1, 2.5:1, 5:1; *n* = 3). Cytolytic activity was evaluated via an LDH release assay. ∗*p* < 0.05 and ∗∗∗∗*p* < 0.0001 by two-way ANOVA, means ± SD. (B) ELISA quantification of mouse IL-2, IFN-γ, and TNF-α (E:T = 5:1, *n* = 3). ∗∗*p* < 0.01, ∗∗∗*p* < 0.001, and ∗∗∗∗*p* < 0.0001 by one-way ANOVA, means ± SD. (C) Scheme of the CT26/Her2 subcutaneous transplantation model in BALB/c mice. (D) Tumor volume (mm^3^ = length × width × height). ∗*p* < 0.05, ∗∗*p* < 0.01, ∗∗∗*p* < 0.001, and ∗∗∗∗*p* < 0.0001 by two-way ANOVA, means ± SEM. (E) Kaplan-Meier survival curves of the mice (a volume ≥2,000 mm^3^ was considered the terminal endpoint, *n* = 5). Significance is marked as m9E10-IgG4m + 4D5-Myc-121 vs. m4D5/121 CAR by the log rank (Mantel-Cox) test. ∗∗∗∗*p*＜0.0001. (F) Quantification of the mouse IL-2 and TNF-α levels on day 10 (*n* = 8). ∗*p* < 0.05, ∗∗*p* < 0.01, and ∗∗∗∗*p* < 0.0001 by one-way ANOVA, means ± SEM. (G) Quantification of mouse IFN-γ levels on days 8 and 14 (*n* = 8). ∗*p* < 0.05, ∗∗*p* < 0.01, ∗∗∗*p* < 0.001, and ∗∗∗∗*p* < 0.0001 by one-way ANOVA, means ± SEM. (H) Tumor-infiltrating CAR-T cells (left) and mPD-1^+^ of CAR-T cells (right) (*n* = 6). Significance is marked as m9E10-IgG4m + 4D5-Myc-121 vs. m4D5/121 CAR, m9E10-IgG4m + 4D5-Myc-121 vs. m121/4D5 CAR. ∗*p* < 0.05 and ∗∗*p* < 0.01 by one-way ANOVA, means ± SEM. (I) Immunofluorescence staining analysis of CD31^+^ tumor blood vessels (*n* = 3), CD31 (red), and DAPI (blue). Scale bars, 100 μm. See also Figures S13, S14, and S15.

We then compared the antitumor activity of murine sCAR-T cells to that of conventional tandem CAR-T cells. Murine sCAR-T showed significantly stronger target killing and cytokine secretion than tandem CAR-T *in vitro* (Figures 5A and 5B). In syngeneic tumor models (Figure 5C), sCAR-T effectively suppressed tumor growth, prolonged survival, caused no treatment-related deaths (Figures 5D and 5E), and induced minimal body weight loss (Figure S15A). All murine CAR-T cells *in vivo* were detected by their (G4S)_n_ linker (Figure S15B) to avoid confusion between adoptively transferred CAR-T cells and autologous T cells. On day 8, compared with conventional CAR-T cells, sCAR-T cells induced a significantly greater release of proinflammatory cytokines (Figures 5F and 5G). Further analysis of tumor samples on day 14 revealed that 4D5-Myc-121 sCAR-T exhibited enhanced tumor infiltration (Figures 5H, left; and S15C) and lower PD-1 expression (Figure 5H, right). Furthermore, sCAR-T also normalized tumor vasculature and cut off nutrient supply to tumor cells (Figure 5I). Safety assessment showed no obvious toxicity in the lung, liver, spleen, or heart across all CAR-T groups (Figure S15D). These findings provide further evidence that antibody-ligand motifs mediate the efficacy of sCAR-T cells in the treatment of solid tumors, demonstrating the capacity of these engineered sCAR-T cells to maintain proliferation, resist exhaustion, and persist both *in vitro* and *in vivo*, without treatment-related toxicities, thus substantially improving therapeutic effect.

### Potential of sCAR-T cells to combat immune escape

**Figure 6:**
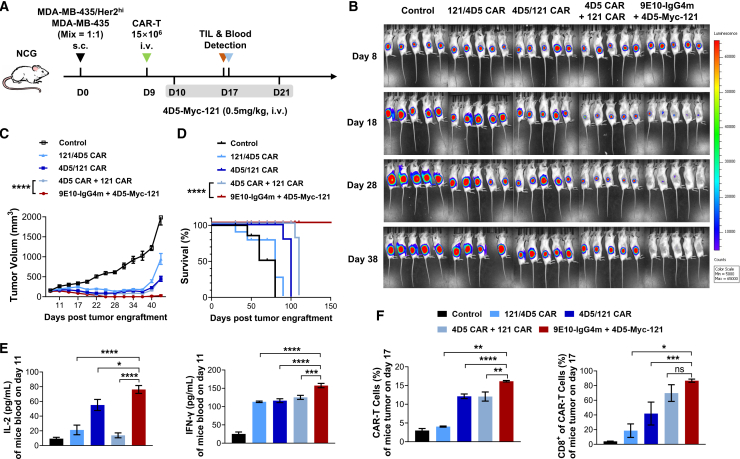
Validation of the efficacy of sCAR-T cells in overcoming immune evasion (A) Schematic showing the establishment of the mixture subcutaneous transplantation model in NCG mice. (B) Representative bioluminescence images of mice subjected to different treatments. The colors represent the luminescence intensity (red, highest; blue, lowest). (C) Tumor volume (mm^3^ = length × width × height). ∗∗∗∗*p* < 0.0001 by two-way ANOVA, means ± SEM. (D) Kaplan-Meier survival curve of the mice (a tumor volume ≥2,000 was considered the terminal endpoint, *n* = 5). Significance is marked as 9E10-IgG4m + 4D5-Myc-121 vs. 4D5 CAR +121 CAR by the log rank (Mantel-Cox) test. ∗∗∗∗*p*＜0.0001. (E) Quantification of human IL-2 and IFN-γ levels 24 h after the first switch from mouse serum (*n* = 7). ∗*p* < 0.05 and ∗∗∗∗*p* < 0.0001 by one-way ANOVA, means ± SEM. (F) Proportion of tumor-infiltrating CAR-T cells (left) and CD8^+^ CAR-T cells (right). ∗*p* < 0.05, ∗∗*p* < 0.01, ∗∗∗*p* < 0.001, and ∗∗∗∗*p* < 0.0001 by one-way ANOVA, means ± SEM. See also Figure S16.

Heterogeneity is a critical factor contributing to immune escape following treatment in solid tumors and is also the primary reason for treatment failure in these malignancies. To evaluate whether sCAR-T cells overcome immune escape, we established heterogeneous tumor models by mixing the MDA-MB-435 and MDA-MB-435/Her2^hi^ cell lines (Figures 6A and S16A). The results demonstrated that sCAR-T cell therapy increased tumor clearance efficiency (Figures 6B, 6C, and S16B) and significantly prolonged survival (Figure 6D), with no considerable alterations in body weight (Figure S16C). Notably, sCAR-T cell therapy resulted in rapid and complete tumor clearance, with no tumor recurrence by day 38. Furthermore, the serum cytokine levels were significantly higher in sCAR-T-treated mice than in other groups (Figure 6E). Analysis of the tumor-infiltrating T cell content on day 17 confirmed that the 4D5-Myc-121 switch efficiently redirected 9E10-IgG4m CAR-T cells into the tumor microenvironment (Figure 6F, left), with a greater proportion of CD8^+^ T cells (Figure 6F, right). A greater proportion of these tumor-infiltrating CAR-T cells exhibited an effector memory phenotype (Figure S16D). sCAR-T cells showed stronger cytotoxicity than conventional tandem CAR-T cells against both Her2-high- and Her2-low-expressing tumor cells. Additionally, while the cytotoxic activity of the combined single-target CAR-T cells against Her2-low-expressing tumor cells was comparable to that of sCAR-T cells, mice treated with the combined single-target CAR-T cells exhibited rapid tumor recurrence after 28 days, which was supported by their reduced ability to kill Her2-high-expressing tumor cells (Figure S16E). In conclusion, our findings highlight the superior therapeutic efficacy of the split-design antibody-ligand-based CAR-T cell approach over conventional therapies, especially in terms of its potential application in overcoming tumor heterogeneity.

### ROR1 antigenic combinations in the sCAR-T platform

**Figure 7:**
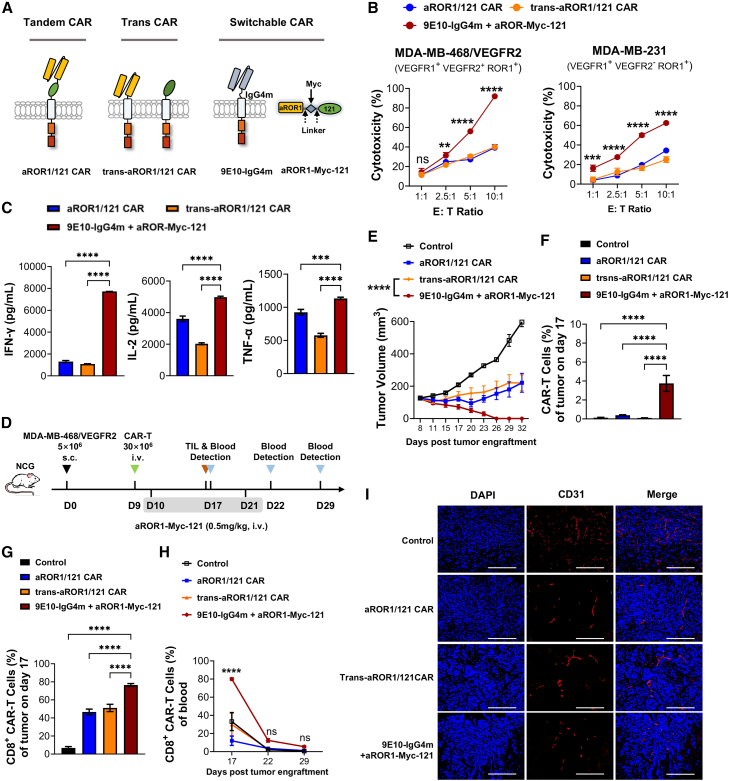
Antitumor efficacy of sCAR-T cells targeting ROR1 (A) Schematic of conventional tandem CAR, transCAR and sCAR structures. (B) Cytotoxicity of CAR-T cells to multiple tumor cell lines. The tumor cells were coincubated with aROR1/121 CAR, trans-aROR1/121 CAR, and 9E10-IgG4m CAR-T cells supplemented with 1 nM aROR1-Myc-121 (E:T ratio = 1:1, 2.5:1, 5:1, 10:1; *n* = 3) for 24 h. Cytolytic activity was evaluated via an LDH release assay. ∗∗*p* < 0.01, ∗∗∗*p* < 0.001, and ∗∗∗∗*p* < 0.0001 by two-way ANOVA, means ± SD. (C) ELISA quantification of human IL-2, IFN-γ, and TNF-α (E:T = 5:1, *n* = 3). ∗∗∗*p* < 0.001 and ∗∗∗∗*p* < 0.0001 by one-way ANOVA, means ± SD. (D) Schematic of the MDA-MB-468 subcutaneous model in NCG mice. (E) Tumor volume (mm^3^ = length × width × height). ∗*p* < 0.05, ∗∗*p* < 0.01, ∗∗∗*p* < 0.001, and ∗∗∗∗*p* < 0.0001 by two-way ANOVA, means ± SEM. (F) Tumor-infiltrating CAR-T cells on day 17 (*n* = 6). ∗∗∗∗*p* < 0.0001 by one-way ANOVA, means ± SEM. (G) Tumor-infiltrating CD8^+^ CAR-T cells (*n* = 6) on day 17. ∗∗∗∗*p* < 0.0001 by one-way ANOVA, means ± SEM. (H) Circulating CD8^+^ CAR-T cells in mouse blood. Significance is marked as 9E10-IgG4m + aROR1-Myc-121 vs. trans-aROR1/121 CAR. ∗∗∗∗*p* < 0.0001 by one-way ANOVA, means ± SEM. (I) Immunofluorescence of CD31^+^ tumor blood vessels (*n* = 2), CD31 (red), and DAPI (blue). Scale bars, 100 μm. See also Figures S17 and S18.

To further evaluate the potential of the antibody-ligand motif-based sCAR-T design for broader application in immunotherapies targeting complex antigen combinations, we extended this strategy to the tumor-associated antigen ROR1. The anti-ROR1 scFv (designated “aROR1”) was derived from an antibody independently screened and isolated in our laboratory (clone: P2-G10), which demonstrated efficient binding to ROR1 (Figure S17A). We constructed the switch molecule aROR1-Myc-121 (Figure S17B) and combined it with 9E10-IgG4m CAR-T cells to generate sCAR-T cells, alongside conventional tandem aROR1/121 CAR and trans-aROR1/121 CAR as controls (Figure S17C). *In vitro* cytotoxicity assays showed that sCAR-T exhibited significantly greater tumor-killing activity and cytokine release than either control CAR design (Figures 7A, 7B, and S17D). To validate *in vivo* efficacy, we established a human breast tumor model by injecting MDA-MB-468/VEGFR2 cells into immunodeficient mice (Figure 7C). sCAR-T treatment achieved complete tumor regression with no recurrence (Figure 7D), accompanied by stable body weight (Figure S17E) and elevated serum proinflammatory cytokines on day 10 (Figure S17F). On day 17, sCAR-T showed deeper tumor infiltration and a higher proportion of CD8^+^ subsets compared with conventional CAR-T cells (Figures 7E and 7F). Whereas the conventional CAR-T groups suppressed tumor growth without complete clearance, sCAR-T cells eradicated tumors by day 26. Therefore, we detected circulating CAR-T cells in the peripheral blood of CAR-T cell-treated mice. Peripheral blood monitoring confirmed higher proportions of total and CD8^+^ CAR-T cells in the sCAR-T group on day 17 (Figures 7G and S18A). By day 22 and 29, CD8^+^ T cells remained more abundant in the sCAR-T group (Figure 7G), although total CAR-T cell frequencies were comparable among groups (Figure S18A). Mechanistically, sCAR-T cells normalized tumor vasculature, disrupting nutrient supply to the tumor cells (Figure 7H). Importantly, no treatment-related toxicity was observed in the lungs, livers, spleens, or hearts (Figure S18B). Together, these results demonstrate that the antibody-ligand motif-based sCAR-T platform effectively integrates antitumor and antiangiogenic therapeutic activities, highlighting its promise for broader application in immunotherapies targeting complex antigen combinations.

## Discussion

Despite the significant strides made in CAR-T cell therapy for hematological malignancies, its efficacy in treating solid tumors remains hampered by the heterogeneity of antigens and the complexities of the tumor microenvironment. To address these obstacles, innovative multitargeting strategies combining antibodies and natural ligands have been developed with promising preclinical and clinical outcomes. In this study, building on prior concepts such as universal CAR-T (uniCAR-T) systems and our earlier work employing APRIL/BAFF ligands in B cell malignancies, we extend this strategy to a tumor/vasculature model by employing structurally optimized antibody-ligand motifs in a split CAR design. This approach allows systematic evaluation of structure-activity relationships and provides a framework for functional exploration of multispecific CAR-T cell therapy. Importantly, this work constitutes the first systematic and comprehensive evaluation of CAR-T cells constructed with antibody-ligand motifs, highlighting their potential to achieve synergistic therapeutic efficacy that overcomes the limitations of conventional CAR-T cell strategies.

Tumor angiogenesis plays a crucial role in tumor progression and immune evasion.^59^ It not only supplies essential nutrients to tumor cells but also creates physical and biochemical barriers that limit T cell infiltration into the tumor microenvironment, thereby reducing the effectiveness of immunotherapy. This dual function makes angiogenesis a highly promising target for improving cancer treatment outcomes.^60^ Combining antivascular and antitumor immunotherapies, such as small-molecule inhibitors, bispecific and trispecific antibodies, and CAR-T cells, has shown significant promise in preclinical studies. Specifically, the combination of bevacizumab and trastuzumab has demonstrated clinical potential against Her2-positive tumors and associated vasculature.^61^^,^^62^^,^^63^^,^^64^^,^^65^^,^^66^ Motivated by these advancements, we aimed to develop a systematic and effective strategy to simultaneously target Her2 and the tumor vasculature by incorporating VEGFR1 and VEGFR2 as complementary angiogenesis-related targets to combat immune evasion and enhance therapeutic efficacy in Her2-positive tumors. Furthermore, we extend the tumor-associated targets to ROR1, demonstrating the broader applicability of this strategy. To increase CAR structural stability and minimize immunogenicity, VEGF-A, a natural ligand for VEGFR1 and VEGFR2, was identified as an optimal candidate. Among the VEGF-A isoforms, VEGF121 stands out for its compact structure, excellent solubility, and heparin-independent binding capacity,^67^ making it widely used in vascular targeting applications such as drug delivery, bioimaging, and therapeutic interventions. In this study, we leveraged VEGF121 and 4D5, a Her2-specific scFv, to construct a trispecific CAR-T system, which demonstrated significant tumor vasculature disruption and robust antitumor effects with minimal off-target toxicity, underscoring the synergy of antivascular and antitumor combination strategies. Given that VEGF121 recognizes both human and murine receptors, we thoroughly evaluated its potential toxicity in tumor-bearing mouse models. Early histological analysis revealed no overt vascular toxicity; however, considering the limited observation period and the occurrence of censored deaths in survival curves, these findings should be interpreted with caution, and a comprehensive evaluation of potential long-term toxicities will require further studies. Collectively, these findings highlight that VEGF121 can serve as a synergistic tool for CAR-T cell therapies targeting solid tumors, playing a significant role in overcoming the current challenges in this field, including those related to the tumor vasculature and immune tolerance.

Solid tumors are challenging to treat with CAR-T cell therapy, primarily due to antigen loss and heterogeneity, often resulting in poor clinical responses.^68^ While dual- or triple-targeted scFv-based CAR-T cells have shown promise in preclinical studies, their development is hindered by chain mispairing, heterogeneous CAR expression, and steric hindrance of multiple binding domains. Replacing the scFv with a natural ligand as the antigen-binding domain has emerged as a viable solution to address the concerns described earlier. Monoclonal antibodies offer high specificity and affinity toward a single epitope, enabling precise target recognition, but their applicability is restricted in heterogeneous or dynamically evolving tumor environments. In contrast, natural ligands—such as cytokines or growth factors—can engage multiple receptors within a molecular family (e.g., BAFF binding to BAFF-R, TACI, and BCMA), providing intrinsic multitargeting capability. However, natural ligands often display lower affinity, poorer tumor selectivity, and dimerization-related off-target risks, requiring structural optimization.^69^ To explore this potential, we adopted an antibody-ligand tandem CAR design to ensure homogeneous expression, but *in vitro* assays showed suboptimal cytotoxicity. The rigidity of the tandem structure constrains VEGF121 near the cell membrane, impairing its natural trans-dimer formation and binding capacity and inhibiting the antigen recognition and signal transduction abilities of the 4D5 scFv. Our previous studies demonstrated that split CAR-T preserves the natural conformation of APRIL and BAFF, significantly enhancing the efficacy of ligand-based CAR-T against B cell malignancies. Building on this concept, we designed an antibody-ligand combination targeting Her2/VEGFR1/VEGFR2 for synergistic sCAR-T therapy. Using a Myc tag as a modular component, we constructed three antibody-ligand motifs—4D5-121-Myc, 4D5-Myc-121, and Myc-4D5-121—to explore various spatial arrangements between the Myc-specific scFv and antigen-binding domains, resulting in distinct scenarios where an artificial IS either formed effectively, mimicking physiological membrane spanning, or failed to form because of overly distal or proximal epitope binding. Among these designs, 4D5-Myc-121 maintained optimal structural distances between either the 4D5 scFv or VEGF121 and T cells (within 10–15 nm) and promoted greater IS formation between target cells and CAR-T cells, therefore inducing highly effective tumor eradication in various tumor models, including a heterogeneous Her2-expressing tumor model. Collectively, these findings highlight that spatial arrangement and distance determine cytotoxicity and efficacy, supporting rational design of multitarget CAR-Ts against solid tumor heterogeneity and antigen loss.

In the context of sCAR-T technology, high-dose administration is often employed to achieve effective *in vivo* tumor suppression. Previous studies including uniCAR-T systems have used 3 × 10^7^ CAR-T cells to achieve therapeutic efficacy. In our *in vivo* experiments, the determination of dosing was guided by the transduction efficiency of the CAR construct (9E10-IgG4m CAR: 32%), which directly informs the distinction between the “injected dose” and the “effective functional dose.” Specifically, 3 × 10^7^ injected cells corresponded to approximately 9.6 × 10^6^ functional CAR-T cells *in vivo*. Similarly, an injected dose of 1.5 × 10^7^ cells resulted in an effective functional population of roughly 4.8 × 10^6^ CAR-T cells. To optimize dosing, we conducted systematic experiments to evaluate the relationship between CAR-T cell doses and tumor suppression efficacy. Based on the comparative analysis of antitumor outcomes across different dose groups, we selected 3 × 10^7^ and 1.5 × 10^7^ cells as the therapeutic doses for subsequent studies. This selection followed a standardized experimental workflow: dose-gradient testing, comparative efficacy assessment, and determination of the optimal therapeutic dose.

In summary, we introduce a split-designed CAR-T cell that targets Her2 and VEGFR1/2, which integrates an innovative and optimized antibody-ligand combination to effectively redirect T cells to solid tumors. This design combines antibodies and ligands for precise target recognition. Notably, sCAR-T promotes IS formation, enhances antitumor activity, prolongs mouse survival, and shows high clinical translation potential. The antibody-ligand combination directs CAR-T accumulation and disrupts tumor vasculature. While disruption of tumor blood vessels may induce local hypoxia and nutrient deprivation, which could be expected to hinder T cell infiltration, our observations suggest that the structural and chemotactic properties of sCAR-T cells, along with changes in chemokine gradients and vascular remodeling, may promote T cell migration into the tumor microenvironment.^70^^,^^71^ This dual mechanism not only increases T cell infiltration but also removes the nutrient supply to tumor cells, working synergistically to suppress tumor growth and effectively overcome immune evasion in solid tumors. Therefore, our sCAR-T cell strategy has high flexibility, effective multitarget recognition ability, and the potential to reduce immunogenicity, making it a promising candidate for the treatment of heterogeneous solid tumors and expanding the possibilities for personalized therapeutic applications.

### Limitations of the study

This study has several limitations. Our primary objective was to develop a multispecific CAR-T system capable of targeting both tumor antigens and the microenvironment vasculature. This study highlights the split design and structural optimization for sCAR-T platform development. Although sCAR-T cells exhibited higher activity (including IFN-γ secretion) than tandem CAR and transCARs, this may not always be beneficial. In particular, increased responsiveness at low antigen densities raises concerns regarding potential on-target/off-tumor reactivity toward healthy tissues. Therefore, higher sCAR-T activity should be interpreted carefully and is not always advantageous. In repeated antigen stimulation assays, sCAR-T cells showed lower Annexin V^+^ fractions compared with conventional CAR-T cells; however, apoptosis across all constructs displayed a non-monotonic pattern. This likely reflects a methodological limitation, as apoptosis was assessed only in viable cells, potentially underestimating late-stage cell death. Accordingly, conclusions regarding apoptosis-related proliferative advantages remain preliminary. Finally, the lack of systematic *in vivo* analyses of immune dynamics, organ toxicity, or behavior limited the safety evaluation. Further preclinical studies are needed to optimize activity thresholds and define sCAR-T translational safety.

## Resource availability

### Lead contact

Further information and requests for resources and reagents should be directed to and will be fulfilled by the lead contact, Dr. Yu J. Cao (joshuacao@pku.edu.cn).

### Materials availability

The materials and reagents used in this study are listed in the key resources table. Reagents generated in this study, including human and murine CAR-T vectors and antibody-ligand motif vectors, will be available upon request. Materials that can be shared will be released via a material transfer agreement.

### Data and code availability

• Full-length sequence data for the newly designed CAR-encoding vectors, antibody-ligand motif plasmids, prediction files of ligand-antibody-based switch plasmids, and the complete AlphaFold server prediction results (including .pdb structural coordinates and .json confidence metrics) are publicly available in Mendeley data and can be accessed via doi:10.17632/4gv2rftv3y.1.
• This study did not generate original code. All protein structure predictions were performed using the AlphaFold 3 Server (https://alphafoldserver.com/). Any custom scripts used for data analysis or plasmid map visualizations are available from the lead contact upon reasonable request.
• Any additional information required to reanalyze the data reported in this paper will be shared by the lead contact upon request.

## Acknowledgments

The authors are deeply grateful to all members of the Y.J.C. group for their technical assistance and helpful discussions. The authors would like to thank Dr. Jialing Zou for his critical discussion in structure prediction. This work was supported by the Shenzhen Medical Research Fund (B2402027), the National Natural Science Foundation of China (32171464 and 81872783), Shenzhen Fundamental Research Program (Natural Science Foundation) - Key Basic Research Project (JCYJ20241202125200001), and the National Key R&D Program of China (2019YFA0906100 and 2019YFA0904200).

## Author contributions

Conceptualization, Y.J.C. and X. Wang; methodology, X. Wang, S.L., and J.G.; investigation, X. Wang, S.L., Q.G., L.S., J.G., X.Q., X. Wei, Q.D., F.H., K.O., and Y.X.; writing – original draft, Y.J.C. and X. Wang; writing – review and editing, Y.J.C., X. Wang, and S.L.; funding acquisition, Y.J.C.; supervision, Y.J.C.

## Declaration of interests

Peking University Shen Zhen Graduate School has filed two patents for this and related technology. Y.J.C., X. Wang, J.G., and L.S. are co-inventors on the patent (202210488015.1; “The combination of triple-target switchable CAR immune cells and its application”). Y.J.C. and X. Wei are co-inventors on the patent (202310705722.6; “Anti-human ROR1 antibodies, genes, and their applications”).

## Declaration of generative AI and AI-assisted technologies in the writing process

During the preparation of this work, the authors used AlphaFold3 Sever in order to predict the structure of switches and CAR structures. After using this tool or service, the authors reviewed and edited the content as needed and take full responsibility for the content of the publication.

## STAR★Methods

### Key resources table

**Table:** 

REAGENT or RESOURCE	SOURCE	IDENTIFIER
**Antibodies**		

Anti-CD3ζ	Santa Cruz Biotechnology	Cat#sc-1239; RRID: AB_627020
anti-phosphorylated CD3ζ	Santa Cruz Biotechnology	Cat#sc-135759; RRID: AB_2073165
anti-VEGFR1	Proteintech	Cat#13687-1-AP; RRID: AB_10644337
anti-VEGFR2	Boster	Cat#A00901-2; RRID: AB_3081281
anti-GAPDH	HUABio	Cat#ET1601-4; RRID: AB_3069615
anti-mouse CD3 antibody	BioLegend	Cat#100301; RRID: AB_312666
anti-mouse CD28 antibody	BioLegend	Cat#102121; RRID: AB_2810330
anti-human PKC-θ biotin-conjugated antibody	Signalway Antibody	Cat#C33151;
anti-CD31 antibody	Servicebio	Cat#GB11063-2; RRID: AB_2922436
PE-conjugated streptavidin	BioLegend	Cat#405204
CY3 goat anti-rabbit IgG	Servicebio	Cat#GB21303; RRID: AB_2861435
pan-Akt antibody	Abcam	ab8805; RRID: AB_306791
phosphorylated -Akt antibody (Ser473)	Cell signaling	#4060; RRID: AB_2315049
Erk1/2 antibody	Abcam	ab17942; RRID: AB_2297336
phosphorylated -Erk1/2 (Thr202/Tyr204)	Cell signaling	#9101; RRID: AB_331646

**Bacterial and virus strains**		

Rosetta gami (DE3) pLysS	BBI	Cat#B528416
*DH5α*	BBI	Cat#A338951

**Biological samples**		

Human peripheral blood mononuclear cells	LEDBIO	Cat#20211112302

**Chemicals, peptides, and recombinant proteins**		

Versene	Gibco	Cat#15040066
Lipofectamine 2000	Invitrogen	Cat#11668-019
CD3/CD28 Dynabeads	Gibco	Cat#11141D
Human IL-2	GenScript	Cat#Z00368-50
Human IL-7	GenScript	Cat#Z02704
Human IL-15	GenScript	Cat#Z03308
Novonectin	Novoprotein	Cat#GMP-CH38
Polybrene	Merck	Cat#H9268
PEI MAX	Polysciences	Cat#24765-1
β-mercaptoethanol	Sigma‒Aldrich	Cat#516732
Ampicillin	BBI	Cat#A430258
Chloramphenicol	BBI	Cat#A600118
Kanamycin	BBI	Cat#A430277
β-D-thiogalactoside	BBI	Cat#A600168
Ni-NTA His-Tag Purification Agarose	Solarbio	Cat#P2010
Thrombin	Solarbio	Cat#T8021-1000U
Tween 20	BBI	Cat#A600560-0500
DAPI	Sigma‒Aldrich	Cat#28718-90-3
Protease inhibitor	APE×BIO	Cat#K4002
Isoflurane	RWD	Cat#R510-22-16
Collagenase	Sigma	Cat#2674
Hyaluronidase	Sigma	Cat#H-3506
DNase I	Sigma	Cat#DN-25

**Deposited data**		

Trastuzumab Fab-Her2 complex	PDB	1n8z
VEGFA-VEGFR1 Complex	PDB	5t89
VEGFA-VEGFR2 complex	PDB	3v2a

**Experimental models: cell lines**		

SK-BR-3	American Type Culture Collection	Cat#HTB-30; RRID: CVCL_0033
SK-OV-3	American Type Culture Collection	Cat#HTB-77; RRID: CVCL_0532
BT474	American Type Culture Collection	Cat#HTB-20; RRID: CVCL_VL01
MDA-MB-435	American Type Culture Collection	Cat#HTB-129; RRID: CVCL_0417
MDA-MB-468	American Type Culture Collection	Cat#HTB-132; RRID: CVCL_0419
HCC1954	American Type Culture Collection	Cat#CRL-2338; RRID: CVCL_1259
NCI-N87	American Type Culture Collection	Cat#CRL-5822; RRID: CVCL_1603
Human umbilical vein endothelial cell line	American Type Culture Collection	Cat#PCS-100-010; RRID: CVCL_A4BC
Porcine aorta endothelial cell line	Sigma-Aldrich	Cat#P30405; RRID: CVCL_UY89
Phoenix Eco	Thermo Fisher Scientific	RRID: CVCL_H717
HEK293-T	Thermo Fisher Scientific	RRID: CVCL_0063
4T1	American Type Culture Collection	Cat#CRL-2539; RRID: CVCL_0125
CT26	American Type Culture Collection	Cat#CRL-2638; RRID: CVCL_7254
murine T cell	This paper	
Jeko-1	American Type Culture Collection	Cat#CRL-3006; RRID: CVCL_1865
MEC-1	American Type Culture Collection	Cat#CRL-3243; RRID: CVCL_1870
MDA-MB-231	American Type Culture Collection	Cat#CRM-HTB-26; RRID: CVCL_0062

**Experimental models: organisms/strains**		

mouse (NOD/ShiLtJGpt-*Prkdc*^*em26Cd52*^*Il2rg*^*em26Cd22*^/Gpt)	GemPharmatech Co., Ltd.	N/A
mouse (BALB∕cAnNCrl)	Charles River Laboratories	N/A

**Recombinant DNA**		

pCL-Eco	Addgene	Cat#12371; RRID: Addgene_12371
pMDLg/pRRE	Addgene	Cat#12251; RRID: Addgene_12251
pRSV-rev	Addgene	Cat#12253; RRID: Addgene_12253
pVSV-G	Addgene	Cat#138479; RRID: Addgene_138479
Lentivirus express vector	Rodgers et al.	N/A
Retrovirus express vector	SnapGene	N/A
4D5 CAR	This paper	doi:10.17632/4gv2rftv3y.1
121-BBz CAR	This paper	doi:10.17632/4gv2rftv3y.1
4D5/121-BBz CAR	This paper	doi:10.17632/4gv2rftv3y.1
121/4D5-BBz CAR	This paper	doi:10.17632/4gv2rftv3y.1
*trans*-4D5/121-BBz CAR	This paper	doi:10.17632/4gv2rftv3y.1
aROR1-BBz CAR	This paper	doi:10.17632/4gv2rftv3y.1
aROR1/121-BBz CAR	This paper	doi:10.17632/4gv2rftv3y.1
*trans*-aROR1/121-BBz CAR	This paper	doi:10.17632/4gv2rftv3y.1
m4D5-28BBz CAR	This paper	doi:10.17632/4gv2rftv3y.1
m4D5/121-28BBz CAR	This paper	doi:10.17632/4gv2rftv3y.1
m121/4D5-28BBz CAR	This paper	doi:10.17632/4gv2rftv3y.1
m121-28BBz CAR	This paper	doi:10.17632/4gv2rftv3y.1
9E10-CD8-BBz CAR	This paper	doi:10.17632/4gv2rftv3y.1
9E10-CD28-BBz CAR	This paper	doi:10.17632/4gv2rftv3y.1
9E10-IgG4m-BBz CAR	This paper	doi:10.17632/4gv2rftv3y.1
m9E10-CD8-28BBz CAR	This paper	doi:10.17632/4gv2rftv3y.1
m9E10-CD28-28BBz CAR	This paper	doi:10.17632/4gv2rftv3y.1
m9E10-IgG4m-28BBz CAR	This paper	doi:10.17632/4gv2rftv3y.1
anti-GD2-28z CAR	This paper	doi:10.17632/4gv2rftv3y.1
4D5/Myc/121	This paper	doi:10.17632/4gv2rftv3y.1
4D5/121/Myc	This paper	doi:10.17632/4gv2rftv3y.1
Myc/121/4D5	This paper	doi:10.17632/4gv2rftv3y.1
4D5/Myc	This paper	doi:10.17632/4gv2rftv3y.1
121/Myc	This paper	doi:10.17632/4gv2rftv3y.1
Myc/121	This paper	doi:10.17632/4gv2rftv3y.1

**Software and algorithms**		

GraphPad Prism 10	Prism	https://www.graphpad.com/features
ImageJ	Image	https://imagej.net/ij/
Living Image	PerkinElmer	https://www.perkinelmer.com.cn/
PyMOL	PyMOL	https://www.pymol.org/
Flowjo	BD	https://www.flowjo.com/solutions/flowjo
FluoView software	Olympus	FV10-ASW version 01.07

**Other**		

AlphaFold3 Sever	Google Deep Mind	https://alphafoldserver.com

### Experimental model and study participant details

#### Mice

Six-to eight-week-old female NOD/ShiLtJGpt-*Prkdc*^*em26Cd5*^*2Il2rg*^*em26Cd22*^/Gpt (NCG) mice purchased from GemPharmatech Co., Ltd. Six-to eight-week-old female BALB/cAnNCrl (BALB/c) mice purchased from Charles River Laboratories.

#### Ethics

All procedures were approved by the Peking University Shenzhen Graduate School Animal Care and Use Committee with approval number AP20231211-01 and were performed according to national and international guidelines for the humane treatment of animals.

#### Cell lines and culture

Human tumor cell lines (SK-BR-3, SK-OV-3, BT474, MDA-MB-435, MDA-MB-231 and MDA-MB-468), human umbilical vein endothelial cell line (HUVEC), porcine aorta endothelial cell line (PAE), and retro- and lentivirus-producing cell lines (Phoenix Eco and HEK293-T) were cultured in Dulbecco’s modified Eagle’s medium (DMEM, HyClone, SH30243.01) supplemented with 10% heat-inactivated FBS (Yoshi, A1015), 1% penicillin‒streptomycin (P/S), 0.1 mM nonessential amino acids (NEAA), 6 mM L-glutamine (L-Glu) and 1 mM sodium pyruvate (SP). Human tumor cell lines (NCI-N87, Jeko-1, MEC-1/ROR1, and HCC1954) and mouse tumor cell lines (4T1 and CT26) were cultured in RPMI-1640 (HyClone, SH30255.01) supplemented with 10% FBS, 1% P/S, 0.1 mM NEAA, 6 mM L-Glu and 1 mM SP. VEGFR2-and/or Her2-positive MDA-MB-468, Her2-positive PAE, Her2-positive CT26 and 4T1, and Her2 highly positive MDA-MB-435 cell lines were generated via lentivirus transduction. The MEC-1/ROR1 cell line was generously provided by Professor Suping Zhang from Shenzhen University. Tumor cell receptor expression was validated by flow cytometry and western blotting (Figure S19).

### Method details

#### Construction of human CAR-encoding vectors and lentivirus production

All CAR structures consisted of the following elements: the EF1α promoter, the CD8 signal sequence, an antigen binding domain, a hinge region, a transmembrane domain, a 4-1BB costimulatory domain, and a CD3ζ signaling domain. The antigen binding domain consisted of either a single-chain variable fragment (scFv) targeting Her2 (4D5, derived from trastuzumab) or ROR1 (P1-G10), in combination with the natural ligand of VEGF121 (designated 121), which binds VEGFR1 and VEGFR2. In these tandem CARs, the scFv and VEGF121 were linked via a flexible GGGGS linker, with either the scFv or VEGF121 positioned proximal to the cell membrane, and all CARs included CD8α hinge and transmembrane regions. For the trans CAR structures, the scFv (4D5 or anti-ROR1) and FLAG-tagged VEGF121 were expressed as separate polypeptides, each containing CD8α hinge and transmembrane regions. The two components were linked via a p2A self-cleaving peptide sequence (GSGATNFSLLKQAGDVEENPGP) to allow coordinated co-expression while preserving the structural integrity and independent function of each domain.

The universal receptor CAR structures included Myc-specific scFv (Clone 9E10) as the antigen binding domain. Specifically, the 9E10-CD8 CAR contained a CD8α hinge region with a CD8α transmembrane domain, the 9E10-CD28 CAR featured a CD28 hinge region with a CD28 transmembrane domain, and the 9E10-IgG4m CAR contained an IgG4m hinge region with a CD8α transmembrane domain.

The sequences were joined via overlap extension PCR and cloned and inserted into the pELPS vector using homologous recombination. The construct was subsequently transformed into DH5α cells for verification via plasmid sequencing.

CAR-expressing viral particles were generated by transfecting HEK293-T cells with lentivirus packaging plasmids (pMDLg/pRRE, pRSV-rev and pVSV-G) and the pELPS plasmid using Lipofectamine 2000 according to the manufacturer’s instructions. Forty-eight hours after transfection, the virus-containing supernatants were collected.

#### Transduction and culture of human CAR-T cells

Human peripheral blood mononuclear cells (PBMCs) were purchased from LEDBIO. PBMCs were thawed and activated with CD3/CD28 Dynabeads for 48 h in X-VIVO 15 (LONZA, 04-418Q) media supplemented with 5% FBS and 300 IU/mL hIL-2. Activated T cells were transduced with the CAR-containing lentivirus in the presence of polybrene, hIL-7 and hIL-15 in novonectin-treated 24-well plates. The plates were centrifuged at 1000 × g for 30 min and then incubated in an incubator. The CD3/CD28 Dynabeads were removed six days after transduction, and the CAR-T cells were expanded to day 14 after activation for subsequent experiments.

#### Construction of murine CAR-encoding vectors and retrovirus production

All murine CAR structures consisted of the following elements: the LCMV promoter, an antigen-binding domain, a hinge region, a transmembrane domain, the CD28 costimulatory domain, the 4-1BB costimulatory domain, and the CD3ζ signaling domain.^27^ The antigen-binding domain of tandem CARs included 4D5 scFv and VEGF121 (codon optimization by GenScript), along with a murine CD28 hinge region and transmembrane domain.^72^ The antigen-binding domain of the universal receptor CARs included a Myc-specific scFv derived from clone 9E10, and the several hinge designs were the same as those used for the human universal receptor CAR structures. The sequences were joined via overlap extension PCR and cloned and inserted into the pTandem vector using homologous recombination. The construct was subsequently transformed into DH5α cells for verification via plasmid sequencing.

CAR-expressing retroviral particles were generated by transfecting Phoenix-Eco cells with the retrovirus plasmid pCL-Eco and the pTandem plasmid using PEI MAX according to the manufacturer’s instructions. Forty-eight hours after transfection, the virus-containing supernatants were collected.

#### Transduction and culture of murine CAR-T cells

Mouse spleens were ground to prepare cell suspensions. A 1 μg/mL anti-mouse CD3 antibody and a 1 μg/mL anti-mouse CD28 antibody were added to activate T cells in RPMI-1640 medium supplemented with 100 IU/mL hIL-2 and 50 μM β-mercaptoethanol for 20 h prior to transduction. Then, the retroviral supernatant was added to novonectin-treated 24-well plates and centrifuged at 2000 × g for 120 min. Then, 1×10^6^ activated murine T cells was added and spun at 600 × g for 10 min. Twenty-four hours after transduction, the medium was replaced with fresh medium supplemented with 100 IU/mL hIL-2 and 50 μM β-mercaptoethanol. Murine CAR-T cells were further expanded for seven days after activation for subsequent experiments.

#### Construction of antibody‒ligand motif switches

The genes encoding the 4D5 scFv, VEGF121 (UniProt: P15692-9), and Myc tag were synthesized by GenScript. The 4D5 scFv, VEGF121 and Myc were attached by various linkers. The flexible GGGS linker was selected to fuse VEGF121 to the C-terminus of the 4D5 scFv, the 218 linker^73^^,^^74^^,^^75^ was selected to attach Myc to the N- or C-terminus of VEGF121, and the (GGGS)_3_ linker was chosen to attach Myc to either the N- or C-terminus of the 4D5 scFv. For the anti-ROR1 construct, the Myc was fused to the C-terminus of the scFv and the N-terminus of VEGF121 to generate aROR1-Myc-121. Additionally, a 6 × His tag was incorporated as a purification marker and separated by a thrombin cleavage site. The sequences were joined via overlap extension PCR and subsequently cloned and inserted into the pET32a(+) vector using homologous recombination. The construct was subsequently transformed into DH5α cells for verification via plasmid sequencing. The verified plasmids were subsequently transformed into *Rosetta gami* (DE3) pLysS cells for protein expression.

#### Prokaryotic expression of antibody‒ligand motif switches

*Rosetta gami* (DE3) pLysS cells were cultured overnight in LB medium and diluted 1:100 in TB medium. The cultures were incubated until the optical density value at 600 nm reached 2.0–2.4, after which isopropyl β-D-thiogalactoside was added to induce expression. The cells were harvested after centrifugation and lysed using ultrasonic disruption. The lysate was centrifuged and filtered. The supernatant was purified using Ni-NTA His-Tag Purification Agarose. The purified protein was dialyzed and cleaved with thrombin and verified by SDS‒PAGE.

#### Purification and detection of switch proteins via size-exclusion chromatography (SEC)

Further purification of native-state switch proteins was performed using SEC. The purified proteins were filtered. Then, the samples were loaded onto a HiLoad 16/600 Superdex 200 pg column (Cytiva, 28989335) and separated using an AKTA pure system operating at a flow rate of 1 mL/min. The native-state switch protein solutions were concentrated, and the endotoxins were removed via anion exchange (PALL, PN MSTG25Q6), sterilized, quantified, and stored at −20°C. For analytical SEC, purified proteins were adjusted to 0.5–1mg/mL, loaded onto a Superdex 200 Increase 10/300 GL column, and run at a flow rate of 0.75 mL/min. Chromatographic peaks were Integrated and analyzed using Unicorn software.

#### ELISA-based binding experiment

The binding affinity of the switch proteins was evaluated by ELISA; 96-well ELISA plates were coated overnight at 4°C with various switch constructs and then blocked. A series of His-tagged recombinant Her2, VEGFR1, or VEGFR2 dilutions were added, and the plates were incubated for 2 h. HRP-labeled anti-His tag antibodies were then added. After the samples were washed with PBS in 0.1% Tween 20, blue color development was performed using a 3,3,5,5-tetramethylbenzidine (TMB) substrate and quantified using a Cytation 5 Cell Imaging Multi-Mode Reader (BioTek) with excitation at 450 nm. The data were plotted and analyzed using GraphPad Prism software.

#### Cytokine release assay

To measure the secretion levels of human and mouse IL-2, TNF-α and IFN-γ, ELISA was carried out using the ELISA kits (human IL-2: Thermo, 88-7024-88; human IFN-γ: Thermo, 88-7314-88; human TNF-α: Thermo, 88-7324-88; mouse IL-2: Thermo, 88-7024-77; mouse IFN-γ: 88-7314-88; mouse TNF-α: 88-7324-88). The samples of the *in vitro and in vivo* experimental groups were harvested after centrifugation at 3000 rpm at 4°C, and then the supplier’s protocol was followed to determine the level of cytokines.

#### Microscopy analysis of IS formation

Immunofluorescence-based conjugation studies were performed using SK-OV-3 cells and CAR-T cells. SK-OV-3 cells were transduced with GFP fluorescent protein. 9E10-CAR-T cells were preincubated with 100 nM protein switches at 4°C for 1 h, following a cold PBS wash, a total of 1 × 10^4^ SK-OV-3 and 2 × 10^4^ CAR-T/sCAR-T cells were coincubated at 37°C before being plated onto poly-L-lysine precoated coverslips. The cells were fixed with paraformaldehyde, permeabilized with 0.2% Triton X-100, and stained with an anti-human PKC-θ biotin-conjugated antibody followed by PE-conjugated streptavidin. The nuclei were stained with Hoechst (Solarbio, C0031). In the final step, a fluoroshield quencher (Sigma‒Aldrich, F6182) was used to prevent fluorescence quenching. Images were acquired using an oil immersion objective (100×) on a laser-scanning confocal microscope (Nikon A1R). Image processing and analysis were conducted using FluoView software (FV10-ASW version 01.07, Olympus) and ImageJ software.

#### Intracellular calcium flux measurement

CAR-T cells were loaded with the calcium indicator Flou4 AM (F14217, Invitrogen) following the manufacturer’s protocol. Time-lapse fluorescence changes, representing intracellular Ca^2+^ flux, were recoded using a Cytation 5 Cell Imaging Multi-Mode Reader (BioTek). For baseline measurement, cells were first collected at 37°C. Subsequently, cells were stimulated with SK-OV-3 cells either alone or in combination with the switch proteins. Fluorescence intensity was monitored over time to assess calcium signaling dynamics during CAR-T cell activation.

#### *In vitro* rechallenge assay

GFP^+^ SK-OV-3 cells were digested and seeded in 24-well plates at a concentration of 5 × 10^5^ per well 12 h prior to the addition of CAR-T cells. CAR-T cells normalized according to transduction efficiency were added at a 2:1 effector-to-target ratio, with the culture supplemented with human IL-7 and human IL-15. On day 3 of coculture, the cells were harvested and analyzed via flow cytometry for CD3 or GFP expression, with 9E10-IgG4m CAR-T cells alone used as a negative control. Twelve hours prior to each round of coculture, SK-OV-3 cells at a concentration of 5 × 10^5^ were added to each well of a 24-well plate, with three replicates per group.

#### *In vitro* cytotoxicity assay

Cytotoxicity assays were performed using human or murine CAR-T cells as the effector cells and tumor cells as the target cells. All target cells were digested by versene (Gibco, 15040066). To assess the cytotoxicity of conventional CAR-T cells, the target cells were mixed with effector cells at different E:T ratios and incubated for 24 h. To assess the cytotoxicity of the sCAR-T cells, the target cells were mixed with the effector cells and incubated with different concentrations of the switch proteins for 24 h. Cytotoxic activity was determined by measuring the lactate dehydrogenase (LDH) levels in the cultured supernatant using the CytoTox 96 Nonradioactive Cytotoxicity Kit (Promega, G1780). T cells were used as a blank control, The percent cytotoxicity was calculated as follows: Cytotoxicity % = 100 × (Experimental LDH Release - Blank)/(Maximum LDH Release - Blank).

#### *In vitro* tube formation assay

24-well plates were precoated with 200 μL of Matrigel matrix per well and allowed to solidify at 37°C for 30 min and HUVECs were seeded into each well. 4D5-Myc-121 was incubated with sCAR-T cells for 1 h, and then CAR-T/sCAR-T cells were added cells per well. The control group was treated with DMEM. The cells were incubated at 37°C with 5% CO_2_ for 6 h to allow migration and tube formation. Images were performed using a Cytation 5 Cell Imaging Multi-Mode Reader. Quantitative analysis of the tube networks according to the number of junctions (nodes) was performed via ImageJ software.

#### Flow cytometry analysis

Surface expression of 4D5 scFv on human and murine CAR-T cells was evaluated using Alexa Fluor 647-conjugated goat anti-human IgG (H + L) in PBS containing 2% FBS. Surface expression of aROR1 scFv on human CAR-T cells was assessed using Alexa Fluor 647-conjugated goat anti-mouse IgG (H + L) under the same conditions. Transduction efficiency of human and murine CAR-T cells expressing 9E10 clone was determined using Alexa Fluor 647-conjugated goat anti-mouse IgG (H + L). For CAR-T cells expressing VEGF121, transduction efficiency was evaluated using VEGFR1-Fc as the primary antibody, followed by Alexa Fluor 647-conjugated goat anti-human IgG (H + L) as the secondary antibody. In trans CAR-T cells, VEGFR121 expression was assessed with PE-conjugated anti-FLAG antibodies. Tumor cell surface expression of Her2 was measured using APC-conjugated anti-Her2 antibodies. Tumor cell surface expression of ROR1 was measured using Alexa Fluor 647-conjugated anti-ROR1 antibodies. For *in vivo* studies, human CAR-T cells were stained with 7-AAD, Pacific Blue- or APC-conjugated anti-human CD3, PE-conjugated anti-human CD8, APC-conjugated anti-human CCR7, FITC-conjugated anti-human CD45RO, and PE-conjugated anti-human PD-1 antibodies. Murine CAR-T cells were stained with APC-conjugated anti-(G4S)_n_ to evaluate CAR expression, and PE-conjugated anti-mouse CD8 and Brilliant Violet 421-conjugated anti-mouse PD-1 antibodies. Flow cytometry was performed on Attune NxT system (Thermo Fisher Scientific), and all data were analyzed using FlowJo software (v10.8, BD Biosciences).

#### Western blot analysis

Proteins were extracted using strong lysis buffer (Mei5bio, MF182-plus-01) supplemented with protease inhibitors. Protein concentrations were determined using a BCA assay (Elabscience, E-BC-K318-M). Equal amounts of protein were resolved on SDS–PAGE gels and separated based on molecular weight via electrophoresis. Proteins were then transferred onto PVDF membranes and blocked. Then incubated overnight at 4°C with primary antibodies. After washing with TBST, membranes were incubated with HRP-conjugated secondary antibodies. Protein bands were visualized using a chemiluminescent HRP substrate and captured with an imaging system. For sequential detection of multiple proteins, membranes were stripped and re-probed following the same procedure.

Primary antibodies included anti-CD3ζ and anti-phospho-CD3ζ for CAR signaling detection; anti-ERK, anti-phospho-ERK, anti-Akt, anti-phospho-Akt, anti-VEGFR1, and anti-VEGFR2 for tumor cell signaling analysis. HRP-conjugated goat anti-mouse IgG and HRP-conjugated goat anti-rabbit IgG were used as secondary antibodies, depending on the primary antibody. GAPDH was used as the loading control for normalization.

#### *In vivo* efficacy study

All studies of human CAR-T cell efficacy were conducted with six-to eight-week-old female NOD/ShiLtJGpt-*Prkdc*^*em26Cd52*^*Il2rg*^*em26Cd22*^*/*Gpt (NCG) mice. The *in vivo* efficacy of human sCAR-T cells compared with that of conventional CAR-T cells was evaluated in the human ovarian cancer cell line SK-OV-3. SK-OV-3 cells (5×10^6^) in a 50% Matrigel (BD Bioscience) solution were subcutaneously implanted into the right flanks of the mice. Nine days after tumor implantation, the mice were administered expanded CAR-T cells via tail vein injection. In particular, the conventional tandem group received 30 × 10^6^ 4D5/121 CAR-T or 121/4D5 CAR-T cells, the transCAR group received 30 × 10^6^ *trans*-4D5/121 CAR-T cells; and the sCAR-T group received 30 × 10^6^ 9E10-IgG4m CAR-T cells combined with 4D5-Myc-121 (0.5 mg/kg, 11 times) via tail vein injection 24 h later. Furthermore, two control groups were established and received only 4D5-Myc-121 (0.5 mg/kg, 11 times) or 9E10-IgG4m CAR-T cells (30 × 10^6^).

In the mixture tumor model, a 1:1 mixture of the human breast cancer cell lines MDA-MB-435 and MDA-MB-435/Her2^hi^ (a total of 5 × 10^6^) in a 50% Matrigel solution was subcutaneously implanted in the flanks of the mice. Nine days after tumor implantation, the mice were administered expanded CAR-T cells via tail vein injection. In particular, the conventional tandem group received 15 × 10^6^ 4D5/121 CAR-T cells or 121/4D5 CAR-T cells, the single-target sequential group consisted of a 1:1 mixture of 4D5 CAR-T cells and 121 CAR-T cells (a total of 15 × 10^6^), and the sCAR-T group received 15 × 10^6^ 9E10-IgG4m CAR-T cells and was administered 4D5-Myc-121 (0.5 mg/kg, 13 times) via the tail vein 24 h later. Furthermore, a control group received only 9E10-IgG4m CAR-T cells.

To assess the *in vivo* efficacy of ROR1-targeted sCAR-T cells, MDA-MB-468/VEGFR2 cells were subcutaneously implanted into mice (5 × 10^6^ in 50% Matrigel solution). Nine days after tumor implantation, mice were administered expanded CAR-T cells via tail vein injection. In the conventional CAR groups, mice received 30 × 10^6^ aROR1/121 CAR-T or *trans*-aROR1/121 CAR-T cells. In the sCAR-T group, mice received 30 × 10^6^ 9E10-IgG4m CAR-T cells combined with aROR1-Myc-121 (0.5 mg/kg, 11 times) via tail vein injection 24 h later. Control groups received 30 × 10^6^ 9E10-IgG4m CAR-T cells alone.

The *in vivo* efficacy of murine sCAR-T cells compared with that of murine conventional CAR-T cells was investigated using six-to eight-week-old female BALB/cAnNCrl (BALB/c). To investigate the killing activity of CAR-T-cells against solid tumors in a competent immune system, the mouse colorectal cancer cell line CT26 was used to construct CT26/Her2 cells overexpressing Her2 to establish a murine tumor model. CT26/Her2 cells (2 × 10^6^) were implanted subcutaneously into the right flanks of the mice. Seven days after tumor implantation, the mice were administered 10 × 10^6^ CAR-T cells via the tail vein. In particular, the conventional tandem group received 10 × 10^6^ m4D5/121 CAR-T or m121/4D5 CAR-T cells, and the sCAR-T group received 10 × 10^6^ m9E10-IgG4m CAR-T cells and was administered 4D5-Myc-121 (0.5 mg/kg, 13 times) via the tail vein 24 h later. Furthermore, two control groups that received only 4D5-Myc-121 or m9E10-IgG4m CAR-T cells were established.

After anesthesia with isoflurane, blood was collected from the orbital venous sinus of each mouse. A total of 50 μL of blood was collected from each mouse for subsequent testing. The mice were euthanized and subjected to autopsy experiments. The heart, liver, lung, and spleen tissues and tumor samples were removed, and fixed in paraformaldehyde in a sequential manner. Half of the tumor samples were fixed in paraformaldehyde for section staining, while the other half were minced and digested for a flow cytometry assay. The tumor digestion solution consisted of collagenase, hyaluronidase, and DNase I, digested with gentle shaking. The tumor suspensions were filtered through cell sieves and then centrifugedand the cells were collected for subsequent analysis. Tumor length, width and height were measured every three days using calipers. Additionally, immune escape model mice were monitored every 10 days using a small animal *in vivo* imaging system (PerkinElmer), and the data was quantified as the total radiance [p/s]. The tumor volume was calculated using the following formula: tumor volume (mm^3^) = length × width × height. The total volume was capped at 2000 mm^3^, at which point the animals were euthanized. In some instances, the specified limit was surpassed before the final measurement, resulting in the immediate euthanasia of the mice.

#### Immunofluorescence staining of blood vessels in tumor tissue

Tumor samples were collected for fixation, paraffin embedding, and sectioning. The paraffin-embedded sections were dewaxed and washed with anhydrous ethanol, and finally washed with distilled water. The sections were blocked and then incubated with rabbit anti-CD31 antibody and CY3 goat anti-rabbit IgG, followed by nuclear counterstaining with DAPI. Immunofluorescence observation was performed using a Cytation 5 Cell Imaging Multi-Mode Reader.

#### Immunohistochemical study

Tumor samples were collected, fixed in 4% formaldehyde, and subsequently embedded in paraffin. Immunohistochemistry was performed using an anti-human or anti-mouse CD3 antibody to assess T cell infiltration. Antibody binding was visualized by adding 3,3′-diaminobenzidine (DAB) substrate. The tumor tissues were further counterstained with hematoxylin. Immunohistochemical staining was performed using a Cytation 5 Cell Imaging Multi-Mode Reader.

#### Histological examination

The collected tissues were subsequently fixed, embedded in paraffin, sectioned, and stained with hematoxylin and eosin. These stained sections were then examined using a Cytation 5 Cell Imaging Multi-Mode Reader.

#### Structure prediction

The structures of 4D5-121-Myc, 4D5-Myc-121 and Myc-4D5-121 switches were predicted by AlphaFold through the AlphaFold Server (https://alphafoldserver.com). We replaced 4D5-Myc-121 with known structures of the trastuzumab Fab-Her2 complex (PDB: 1n8z),^76^ the VEGFA-VEGFR1 complex (PDB: 5t89)^77^ and the VEGFA-VEGFR2 complex (PDB: 3v2a)^78^ and created a model of the binding of 4D5-Myc-121 to Her2, VEGFR1 and VEGFR2 that accurately reflected their natural spatial structures. We also predicted the structure of 9E10-IgG4m CAR structure expressed on T cells via the AlphaFold server. Structures were analyzed, and figures were generated by using PyMOL (PyMOL Molecular Graphics System, http://www.pymol.org).

### Quantification and statistical analysis

Statistical analyses were conducted with GraphPad Prism version 10.3.1 software (GraphPad Software, LLC). The data are reported as means ± standard deviation (S.D.) for *in vitro* analysis, the *in vivo* data are presented as the mean ± standard error of the mean (S.E.M.). Significant differences were determined using the one-way analysis of variance (ANOVA) or two-way ANOVA. Kaplan-Meier survival analysis was employed for all survival studies, with group comparisons made using the log rank test. A *p* value < 0.05 was considered as statistically significant. For animal studies, the sample size was predetermined by our prior experiments. Statistical details of experiments were described in Figure Legends.

### Additional resources

No additional resources.

## Supplemental information

Document S1. Figures S1–S19 and Tables S1 and S2

Document S2. Article plus supplemental information
