# Supplementary material for: Design optimization of antibody-ligand motifs to enhance CAR-T redirection activity against solid tumors
Source: Cell Rep Med. 2026 Jun 25;7(7):102885. doi: 10.1016/j.xcrm.2026.102885 (PMC13400168; doi:10.1016/j.xcrm.2026.102885)
Supplement: Document S1. Figures S1–S19 and Tables S1 and S2 [file mmc1.pdf]

Cell Reports Medicine, Volume *7*

# Supplemental information

# Design optimization of antibody-ligand motifs to enhance CAR-T redirection activity against solid tumors

**Xuechun Wang, Shuhong Li, Qiaoru Guo, Licai Shi, Jian Guo, Xuexiu Qi, Xiaoyi Wei, Qingen Da, Fang Huang, Kunfu Ouyang, Yang Xu, Jun Li, and Yu J. Cao**

# Supplementary information

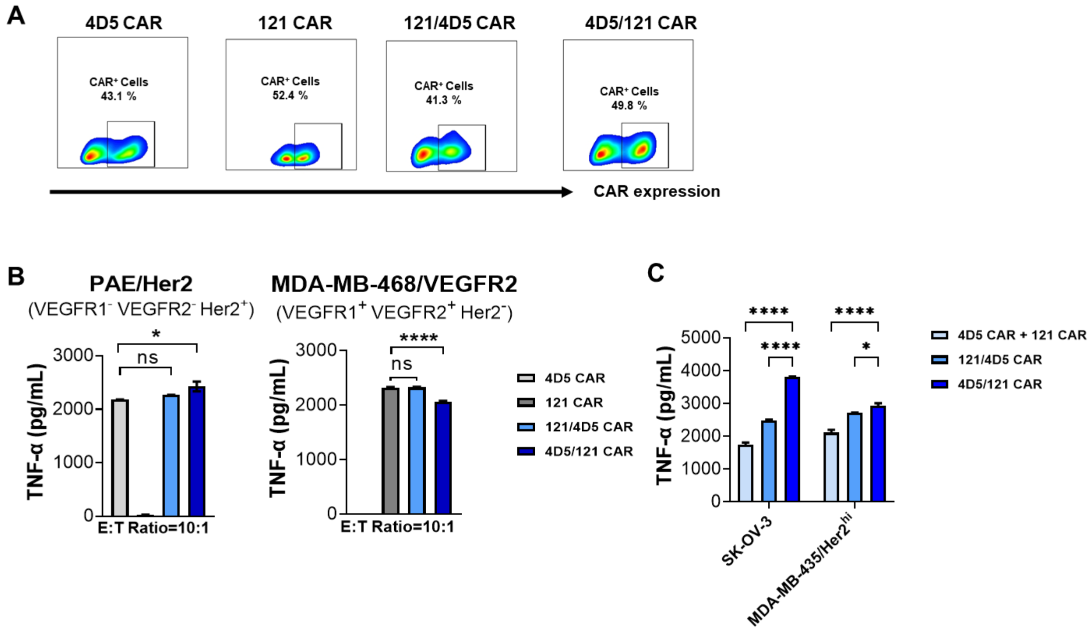


**Figure S1. Antibody–ligand conventional tandem CAR-T-cell efficacy. Related to Figure 1.**

(A) CAR-T cells were detected by flow cytometry and stained with an Alexa Fluor 647-conjugated human IgG antibody to detect the 4D5 scFv level of 4D5 CAR, 4D5/121 CAR and 121/4D5 CAR. The fusion protein VEGFR1-Fc and an Alexa Fluor 647-conjugated human IgG antibody were used to detect the VEGF121 level of 121 CAR. (B) Human TNF-α release of CAR-T cells cocultured with PAE/Her2 (E:T ratio=10:1, n=3) and MDA-MB-468/VEGFR2. 4D5 CAR vs. 121/4D5 CAR, 4D5 CAR vs. 4D5/121 CAR, 122 CAR vs. 121/4D5 CAR and 121 CAR vs. 4D5/121 CAR,*P < 0.05, ****P < 0.0001 and ns=P>0.05 by one-way ANOVA, means ± SDs. (C) Human TNF-α release of CAR-T cells cocultured with SK-OV-3 and MDA-MB-435/Her2$^{hi}$ (E:T ratio=10:1, n=3). 4D5/121 CAR vs. 121/4D5 CAR and 4D5/121 CAR vs. 4D5 CAR + 121 CAR, *P < 0.05 and ****P < 0.0001 by one-way ANOVA, means ± SDs.

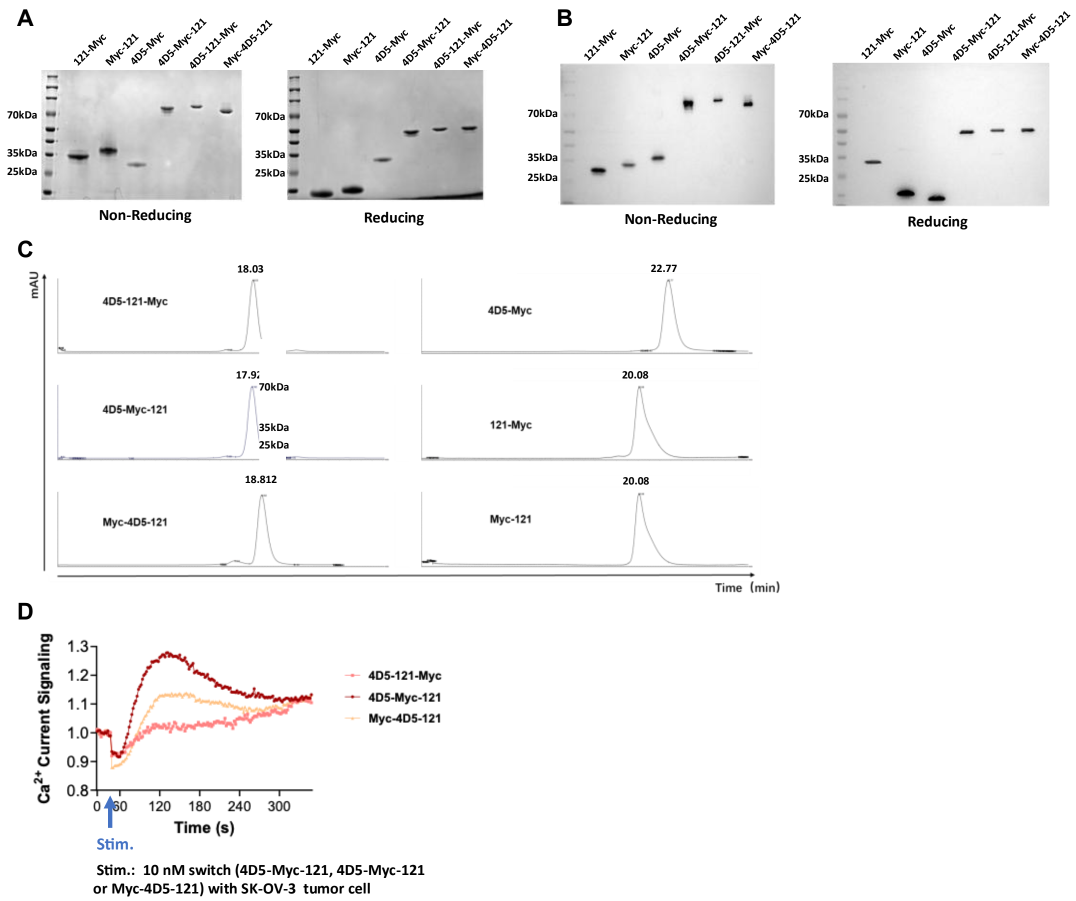


**Figure S2. Antibody–ligand motif-based switch protein validation. Related to Figure 2.**

(A) Purity and molecular weight verification of switches via SDS–PAGE. (B) Western blot analysis of switches using an HRP-conjugated Myc antibody. (C) SEC-HPLC analysis of the switches. (D) $Ca^{2+}$ singling detection with different switches. Intracellular $Ca^{2+}$ signaling was measured in SK-OV-3 tumor cells co-cultured with 9E10-IgG4m CAR-T cells in the presence of 10 nM 4D5-121-Myc, 4D5-Myc-121, or Myc-4D5-121 switches.

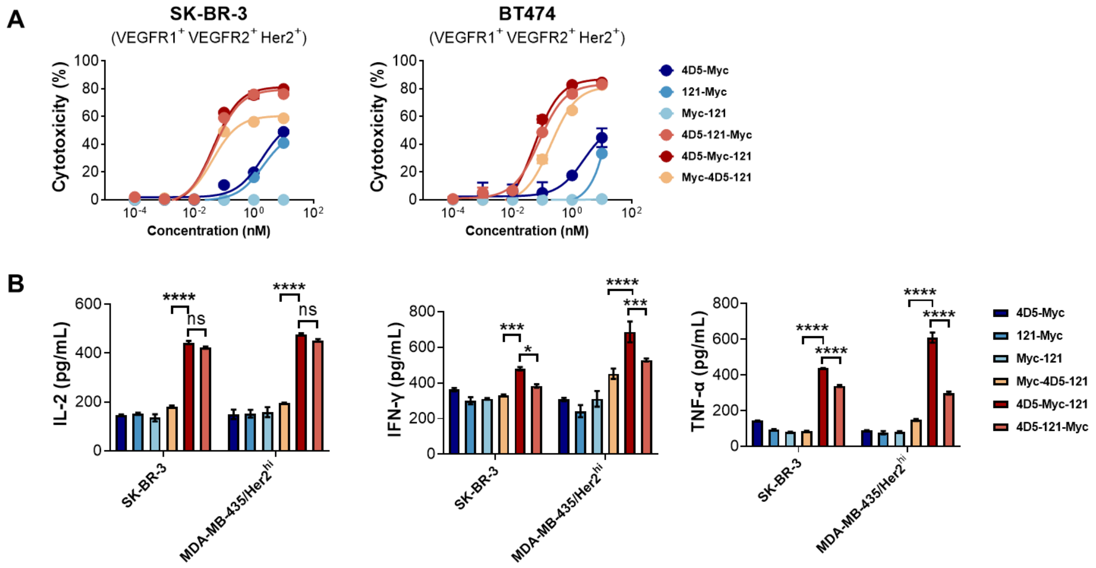


**Figure S3. Optimal efficacy of switch proteins. Related to Figure 2.**

(A) Cytotoxicity of sCAR-T cells to SK-BR-3 and BT474 at an E:T ratio of 10:1 for 24 h (n=3). Cytolytic activity was evaluated via an LDH release assay. (B) Human IL-2, IFN-γ and TNF-α release in sCAR-T cells coincubated with SK-BR-3 and MDA-MB-435/Her2$^{hi}$ at an E:T ratio of 10:1 with different switches (1 nM) was measured by ELISA. (n=3). 4D5-Myc-121 vs. 4D5-121-Myc and 4D5-Myc-121 vs. Myc-4D5-121, *P < 0.05, ***P < 0.001, ****P < 0.0001 and ns=P>0.05 by two-way ANOVA, means ± SD.

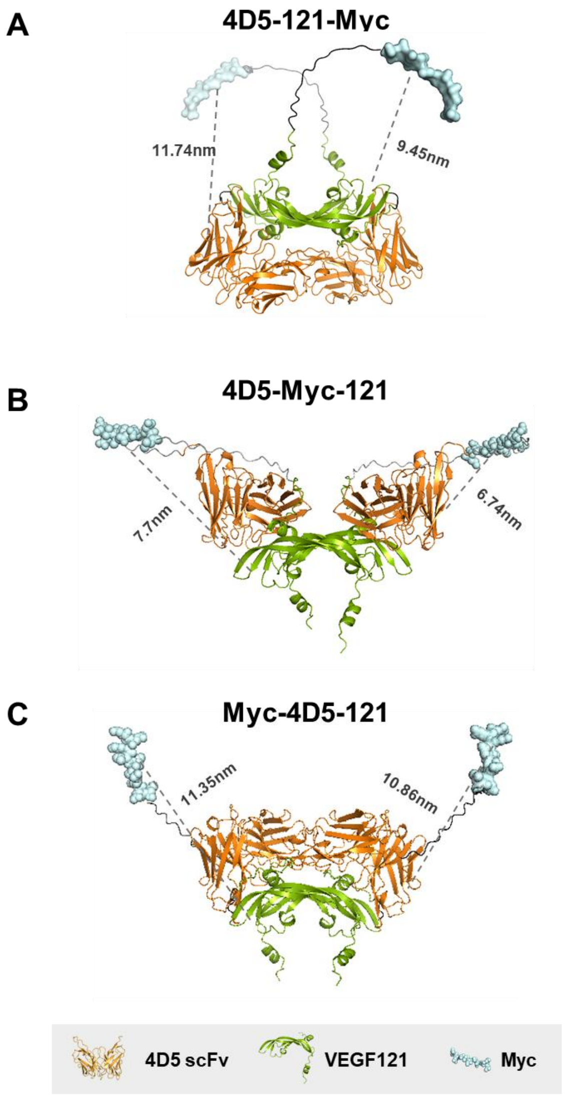


**Figure S4. Prediction of ligand–antibody-based switch fusions via the AlphaFold server. Related to Figure 2.**

(A) Structure prediction of 4D5-121-Myc. The distance between Myc and VEGF121 is 11.74 nm, the distance between Myc and 4D5 scFv is 9.45 nm. (B) Structure prediction of 4D5-Myc-121. The distance between Myc and VEGF121 is 7.7 nm, the distance between Myc and 4D5 scFv is 6.74 nm. (C) Structure prediction of Myc-4D5-121. The distance between Myc and VEGF121 is 11.35 nm, the distance between Myc and 4D5 scFv is 10.86 nm.

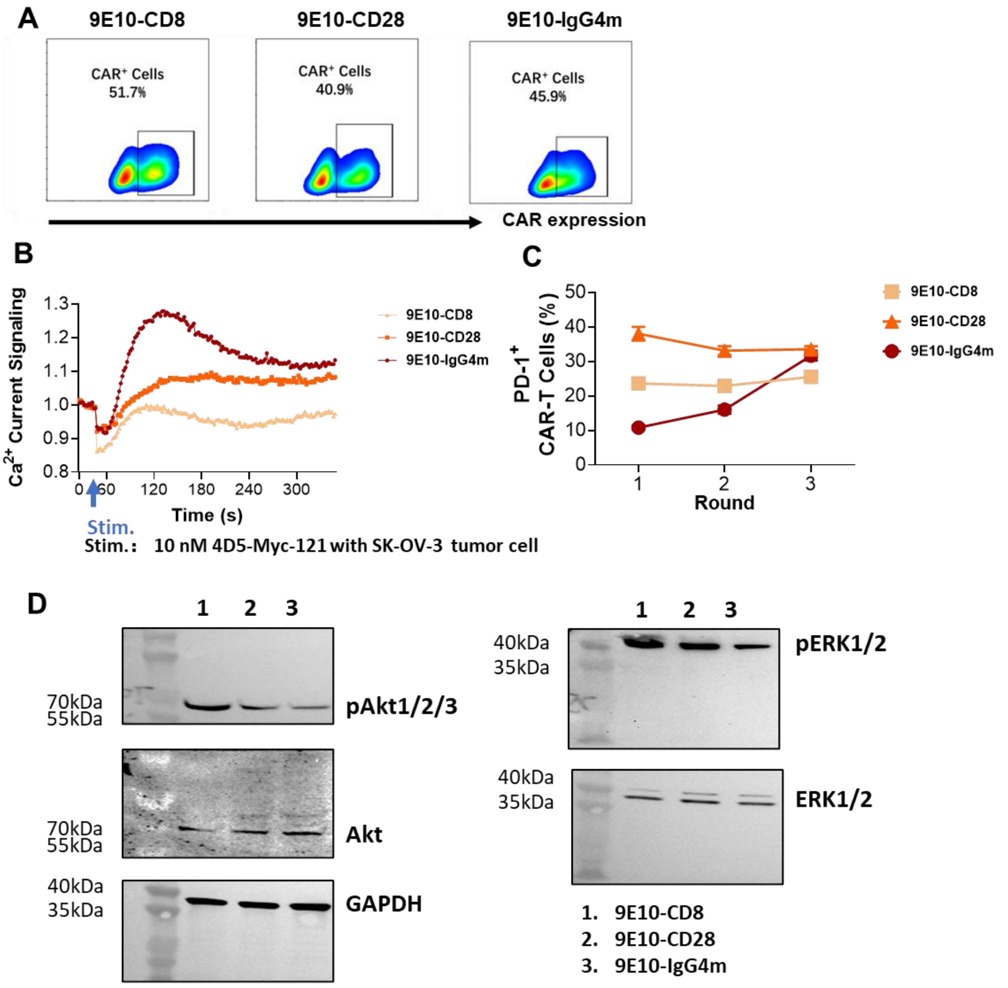


**Figure S5. Optimal hinge of the universal receptor CAR. Related to Figure 3.**

(A) CAR-T cells were detected by flow cytometry and stained with an Alexa Fluor 647-conjugated mouse IgG antibody to detect the 9E10 scFv level in 9E10-CD8, 9E10-CD28 and 9E10-IgG4m CARs. (B) Intercellular $Ca^{2+}$ signaling was assessed in SK-OV-3 tumor cells co-cultured with 9E10-IgG4m CAR-T cells in the presence of 10 nM 4D5-121-Myc, 4D5-Myc-121, or Myc-4D5-121 switches. (C)Proportion of PD-1-CAR-T cells in the rechallenge assay. (D) Western blot analysis of ERK, phosphorylated ERK, Akt, and phosphorylated Akt in lysates from 9E10-CD8 CAR, 9E10-CD28 CAR, and 9E10-IgG4m CAR-T cells.

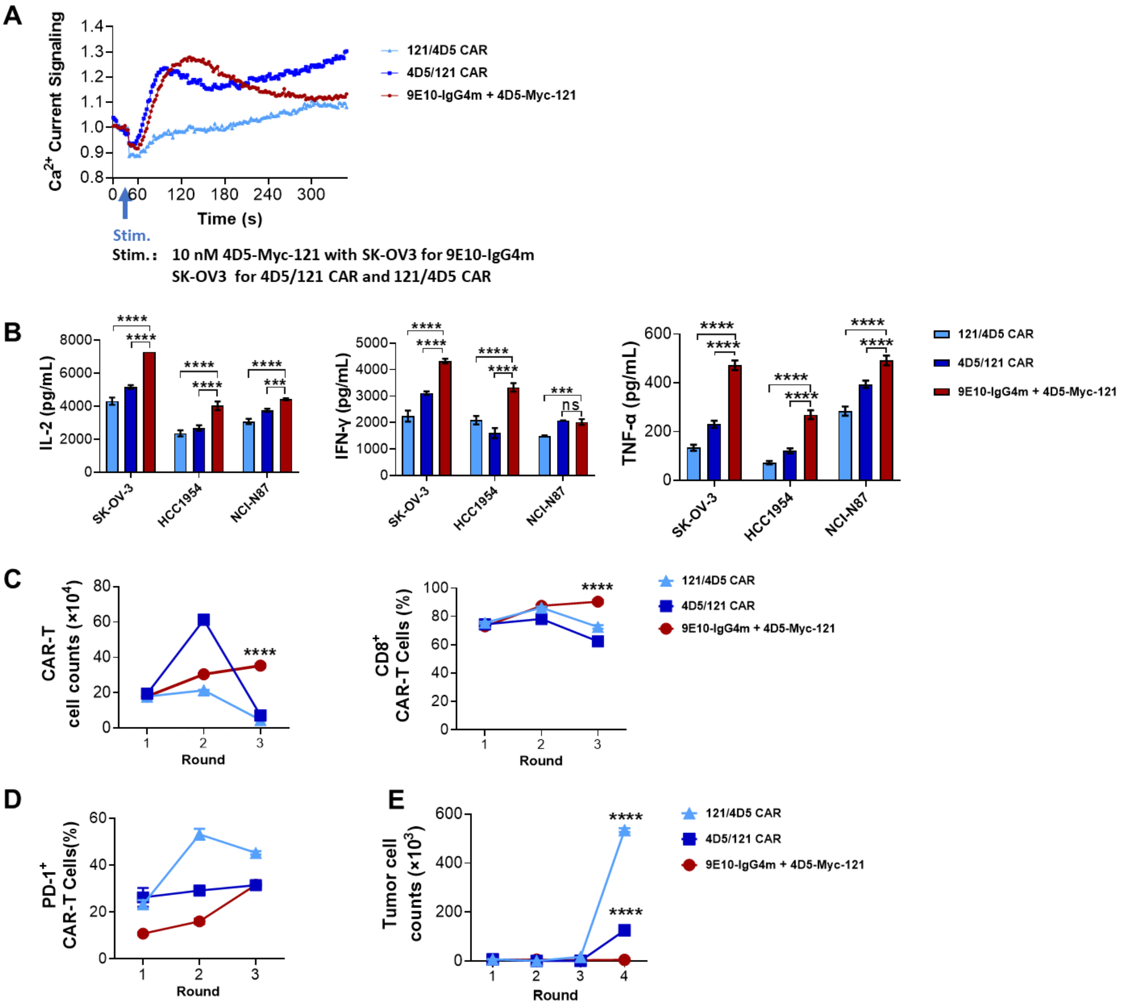


**Figure S6. Comparison of sCAR-T and conventional CAR-T cells *in vitro*. Related to Figure 4.** (A) Intercellular $Ca^{2+}$ signaling was detected with 9E10-IgG4m + 10 nM 4D5-Myc-121, 4D5/121 CAR and 121/4D5 CAR stimulated with SK-OV-3 tumor cells. (B) Human IL-2, IFN-γ and TNF-α detection. Coincubated sCAR-T cells with 1 nM 4D5-Myc-121 or conventional CAR-T cells (4D5/121 CAR or 121/4D5 CAR) cells and SK-OV-3, HCC1954, or NCI-N87 cells at an E:T ratio of 1:1 for 24 h. Cytokine release levels were measured by ELISA (n=3). 9E10-IgG4m + 4D5-Myc-121 vs. 4D5/121 CAR and 9E10-IgG4m + 4D5-Myc-121 vs. 121/4D5 CAR, ***$P < 0.001$, ****$P < 0.0001$ and ns=$P>0.05$ by two-way ANOVA, means ± SD. (C) Quantification of $CD3^+$ cells and $CD8^+$ CAR-T cells in the rechallenge assay. ****$P < 0.0001$ by two-way ANOVA, means ± SD. 9E10-IgG4m + 4D5-Myc-121 vs. 4D5/121 CAR. (D) PD-1 expression in CAR-T cells in a rechallenge assay of sCAR-T cells compared with conventional CAR-T cells. (E) Quantification of tumor cells in a rechallenge assay of sCAR-T cells compared with conventional CAR-T cells. 9E10-IgG4m + 4D5-Myc-121 vs. 4D5/121 CAR and 9E10-IgG4m + 4D5-Myc-121 vs. 121/4D5 CAR, ****$P < 0.0001$ by two-way ANOVA, means ± SD.

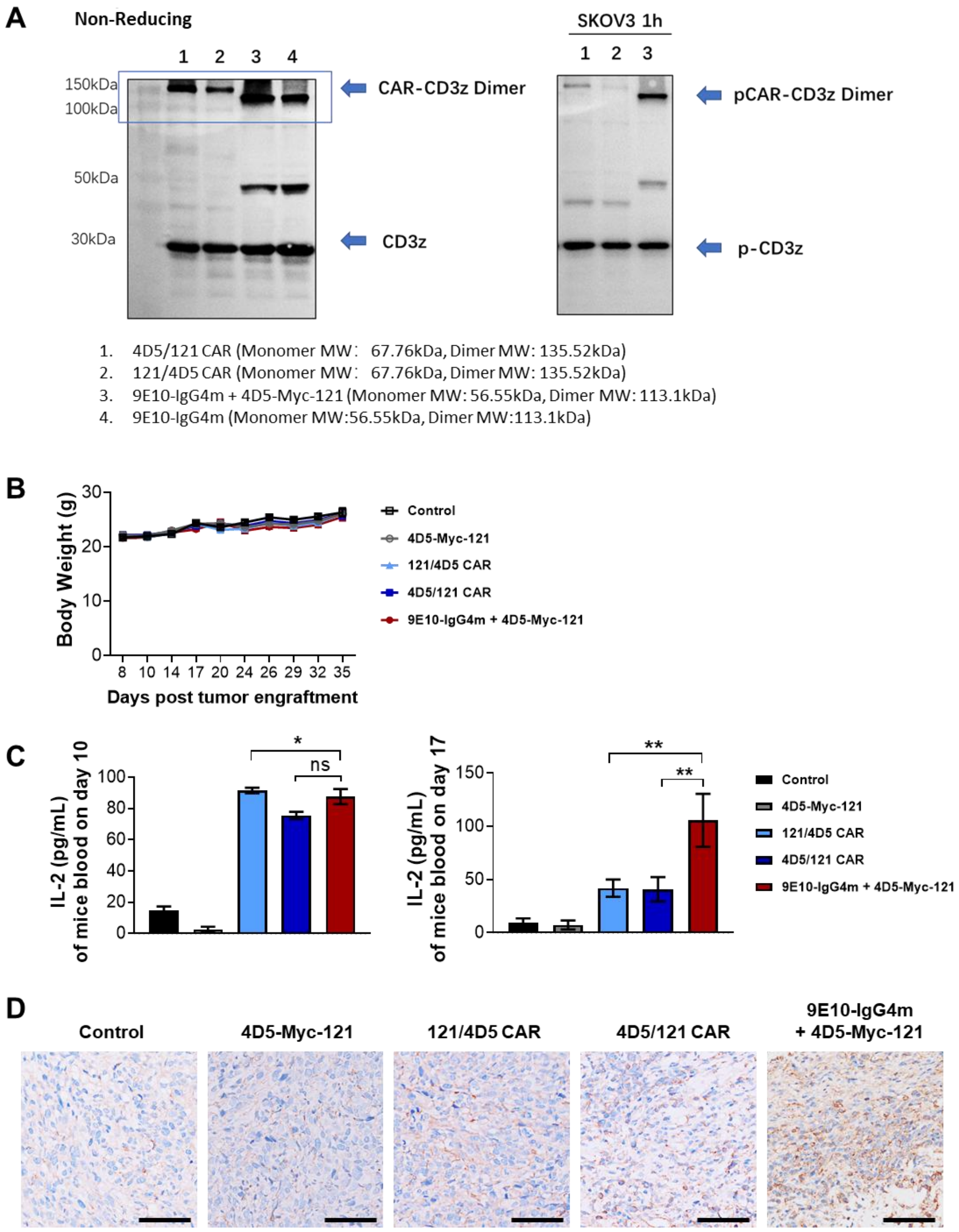


**Figure S7.** ***In vitro*** **CAR-CD3 and CAR-CD3z phosphorylation detection by Western Blot and** ***in vivo*** **validation of sCAR-T. Related to Figure 4.**

(A) CAR-CD3 and CAR-CD3z phosphorylation in 4D5/121 CAR and 121/4D5 CAR-T cells compared with 9E10-IgG4m CAR-T cells + 1 nM 4D5-Myc-121, all groups stimulated with SK-OV-3 cells for 60 min. Lysis of CAR-T cells was performed, and the results were detected by western blotting. (B) Mouse weight changes in each group of SK-OV-3 tumor model mice from the day after CAR-T-cell injection (n=5). (C) Serum levels of human IL-2 on day 10 and day 17 (n=5). $^{**}P < 0.01$ and ns=P>0.05 by one-way ANOVA, means ± SEM. (D) The tumor tissue samples (n=2) were labeled with a mouse anti-human CD3 antibody, and then an HRP-conjugated mouse monoclonal antibody was used as a secondary antibody to amplify the signals. Scale bar, 100 μm.

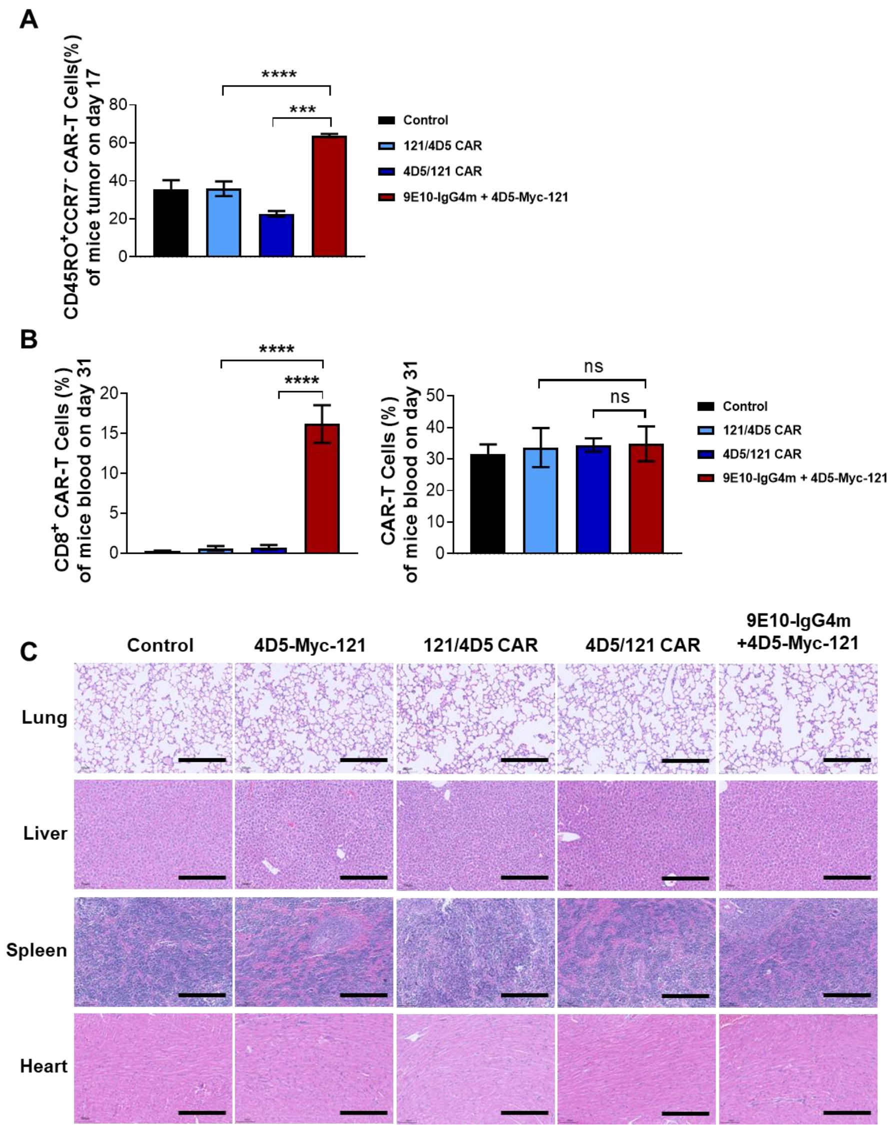


**Figure S8. *In vivo* verification of sCAR-T cells on SK-OV-3 tumor model**. **Related to Figure 4.** (A) Tumor-infiltrating CD45RO$^+$CCR7$^-$CAR-T effect memory cells (n=3) on day 17. ***P < 0.001, and ****P < 0.0001 by one-way ANOVA, means ± SEM. (B) Circulating CAR-T cells (n=5) and circulating CD8$^+$ CAR-T cells (n=5) on day 31. ****P < 0.0001 and ns=P>0.05 by one-way ANOVA, means ± SEM. (C) Hematoxylin and eosin-stained lung, liver, spleen and heart samples from NCG mice in the SK-OV-3 tumor model (n=2) on day 17. Scale bar, 100 μm.

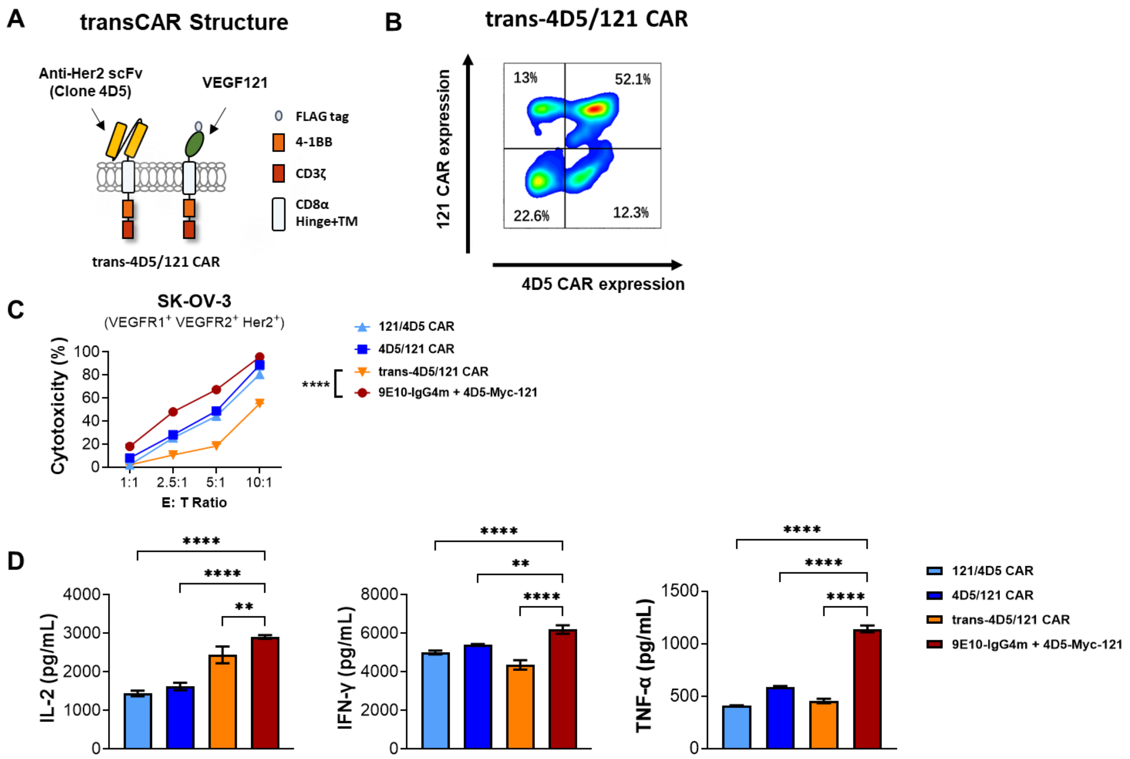


**Figure S9. *In vitro* verification of transCAR-T cell. Related to Figure 4.**

(A) Schematic representation and expression of the trans-4D5/121 CAR. (B) CAR expression of trans-4D5/121 CAR. Alexa Fluor 647-conjugated human IgG antibody were used to detect 4D5 scFv and PE-conjugated FLAG were used to detect FLAG-121. (C) Cytotoxicity of 4D5/121 CAR, 121/4D5 CAR, trans-4D5/121 CAR, and 9E10-IgG4m CAR-T cells supplemented with 1 nM 4D5-Myc-121 against SK-OV-3 tumor cells at indicated E:T ratios (1:1, 2.5:1, 5:1, 10:1; n=3) for 24 h. Cytolytic activity was assessed by LDH release assay. ****P<0.0001 by two-way ANOVA, means ± SD. (D) Human IL-2, IFN-γ and TNF-α release from SK-OV-3 cells co-cultured with 4D5/121 CAR, 121/4D5 CAR, trans-4D5/121 CAR-T cells, or 9E10-IgG4m CAR-T cells plus 1 nM 4D5-Myc-121 at an E:T ratio of 1:1 for 24 h (n=3). Cytokines were quantified by ELISA. **P<0.01 and ****P<0.0001 by one-way ANOVA, means ± SD.

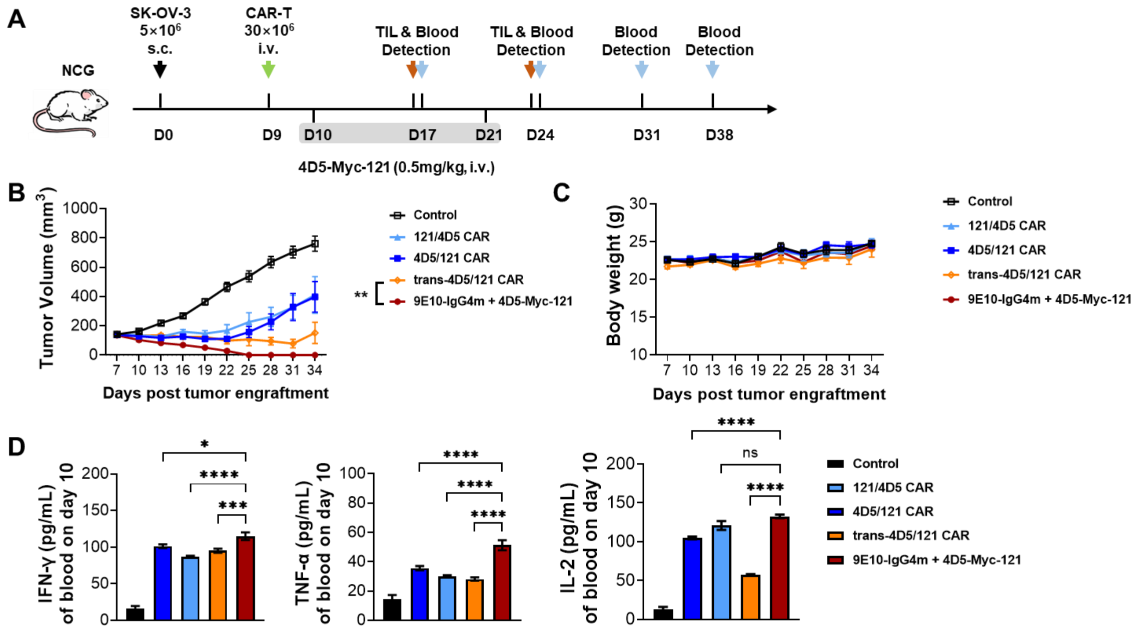


**Figure S10.** ***In vivo*** **validation of sCAR-T cells compared with conventional CARs. Related to Figure 4.**

(A) Experimental timeline. (B) Tumor volume (mm³ = length × width × height) in SK-OV-3 xenograft mice (n=5–7). **P < 0.01 by two-way ANOVA, means ± SEM. (C) Body weight changes in SK-OV-3 tumor–bearing mice following CAR-T cell injection (n=5–9). (D) Serum levels of human IFN-γ, TNF-α and IL-2 on day 10 (n=9). Significance is marked as 4D5/121 CAR vs. 9E10-IgG4m + 4D5-Myc-121, 121/4D5 CAR vs. 9E10-IgG4m + 4D5-Myc-121, trans-4D5/121 CAR vs. 9E10-IgG4m + 4D5-Myc-121, *P<0.05 and ****P<0.0001 by one-way ANOVA, means ± SEM.

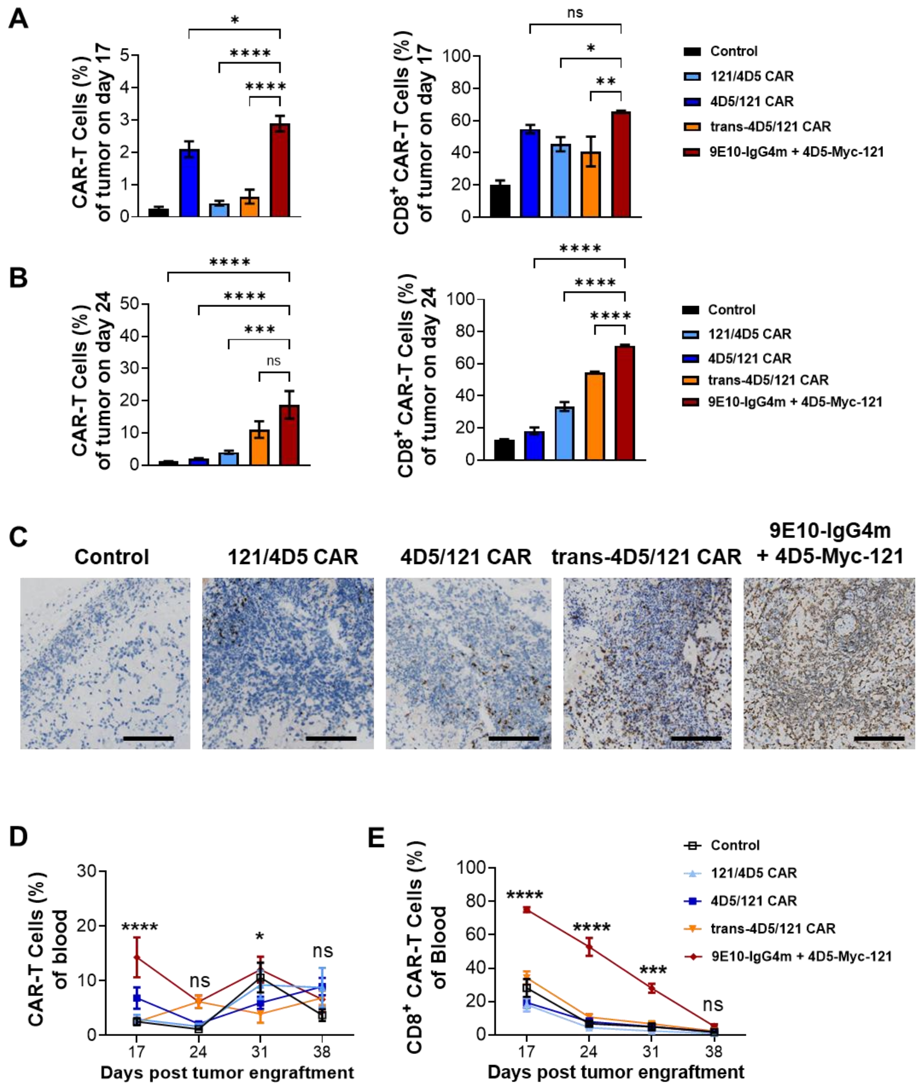


**Figure S11.** ***In vivo*** **validation of sCAR-T cells in the SK-OV-3 tumor model. Related to Figure 4.** (A) Frequency of tumor-infiltrating CAR-T cells (left) and CD8$^+$ CAR-T cells (right) on day 17 (n=6). *P < 0.05, **P < 0.01, ****P < 0.0001 and ns=P>0.05 by one-way ANOVA, means ± SEM. (B) Frequency of tumor-infiltrating CAR-T cells (left) and CD8$^+$ CAR-T cells (right) on day 24 (n=6). ***P < 0.001, ****P < 0.0001 and ns=P>0.05 by one-way ANOVA, means ± SEM. (C) Circulating CAR-T cells in peripheral blood on day 17 (n=5–9). *P < 0.0001, ****P < 0.0001 and ns=P>0.05 by one-way ANOVA, means ± SEM. (D) Circulating CAR-T cells in peripheral blood (n=5–9). Significance is marked as trans-4D5/121 CAR vs. 9E10-IgG4m + 4D5-Myc-121, *P < 0.05***P < 0.001, ****P < 0.0001 and ns=P>0.05 by two-way ANOVA, means ± SEM. (E) Circulating CD8$^+$ CAR-T cells in peripheral blood (n=4). Significance is marked as trans-4D5/121 CAR vs. 9E10-IgG4m + 4D5-Myc-121, ***P < 0.001, ****P < 0.0001 and ns=P>0.05 by two-way ANOVA, means ± SEM.

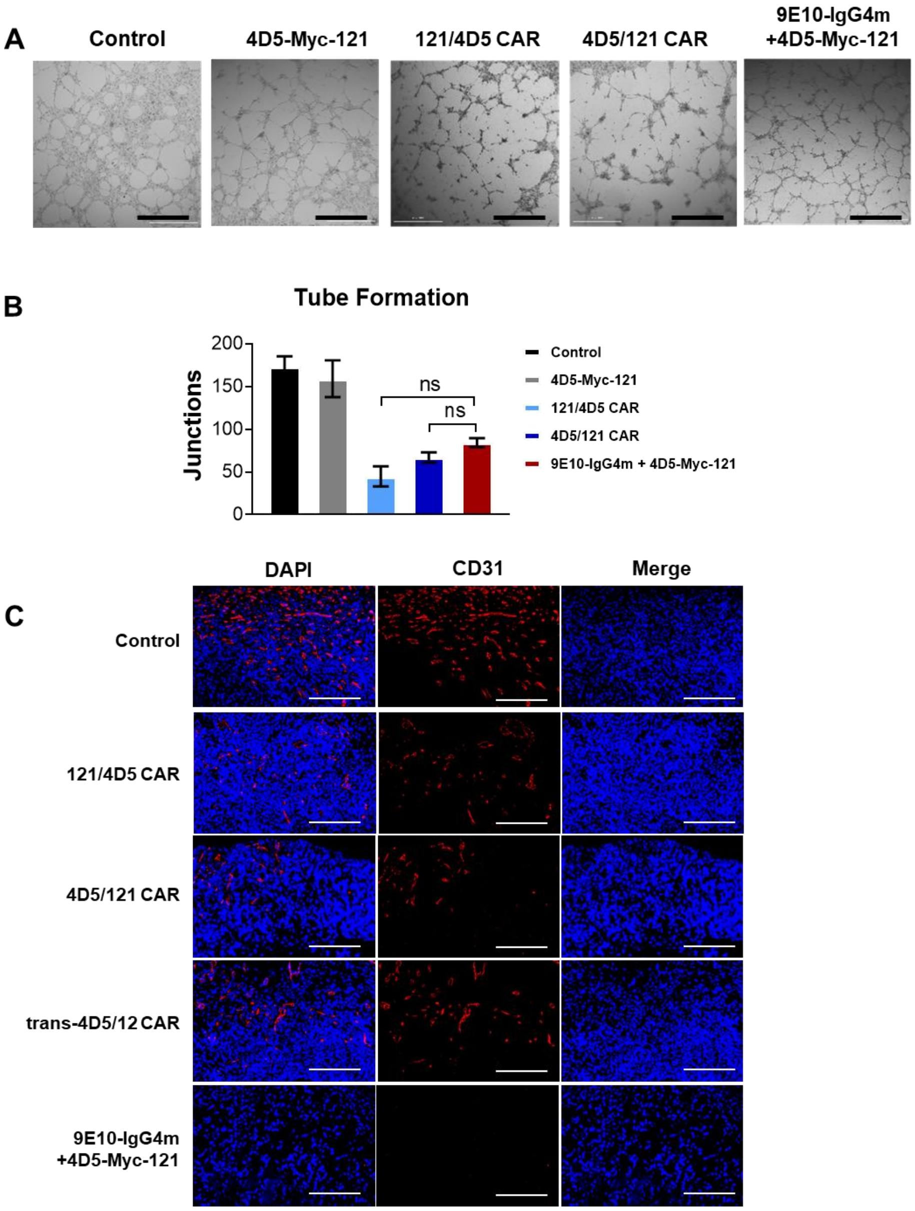


**Figure S12. Tumor vascular analysis. Related to Figure 4.**

(A) An *in vitro* tube formation model was constructed using HUVECs to compare the tube disruption abilities of 4D5/121 CAR, 121/4D5 CAR, and 9E10-IgG4m CAR with 4D5-Myc-121. Scale bar, 1000 µm. (B) Quantitation of tube junctions (n=5). ns=P>0.05 by one-way ANOVA, means ± SEM. (C) Immunofluorescence staining of $CD31^+$ tumor blood vessels in tumor sections (n=2). CD31 (red); nuclei were counterstained with DAPI (blue). Scale bar, 100 µm.

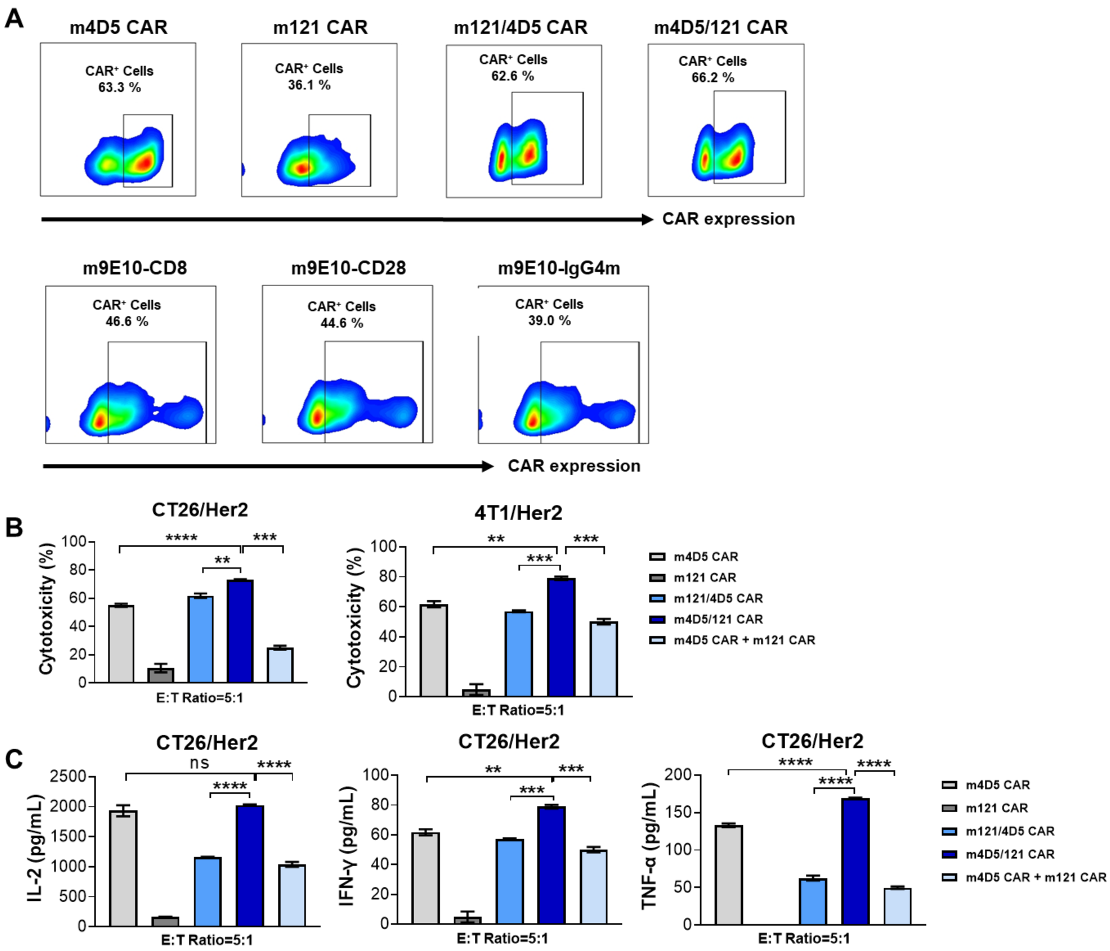


**Figure S13. The expression levels of murine CARs and antibody–ligand conventional tandem murine CAR-T-cell validation. Related to Figure 5.**

(A) CAR-T cells were detected by flow cytometry and stained with an Alexa Fluor 647-conjugated human IgG antibody to detect the 4D5 scFv level expressed by the m4D5 CAR, m4D5/121 CAR or m121/4D5 CAR. The fusion protein VEGFR1-Fc and an Alexa Fluor 647-conjugated human IgG antibody were used to detect VEGF121 expressed by the m121 CAR. An Alexa Fluor 647-conjugated mouse IgG antibody was used to detect m9E10 scFv expressed by the m9E10-CD8, m9E10-CD28 and m9E10-IgG4m CARs. (B) Cytotoxicity of m4D5 CAR, m121 CAR, m4D5/121 CAR, m121/4D5 CAR and the combination of m4D5 CAR and m121 CAR-T cells on the CT26/Her2 and 4T1/Her2. CAR-T cells and target cells were coincubated at an E:T ratio of 5:1 for 24 h. Cytolytic activity was evaluated via an LDH release assay (n=3). **P<0.01, ***P<0.001, ****P<0.0001 and ns=P>0.05 by one-way ANOVA, means ± SDs. (C) Mouse IL-2, IFN-γ and TNF-α detection. m4D5 CAR, m121 CAR, m4D5/121 CAR, m121/4D5 CAR and the combination of m4D5 CAR and m121 CAR-T cells were coincubated with CT26/Her2 at an E:T ratio of 5:1 for 24 h. Cytokine levels were determined via ELISA (n=3). **P<0.01, ***P<0.001, ****P<0.0001 and ns=P>0.05 by one-way ANOVA, means ± SDs.

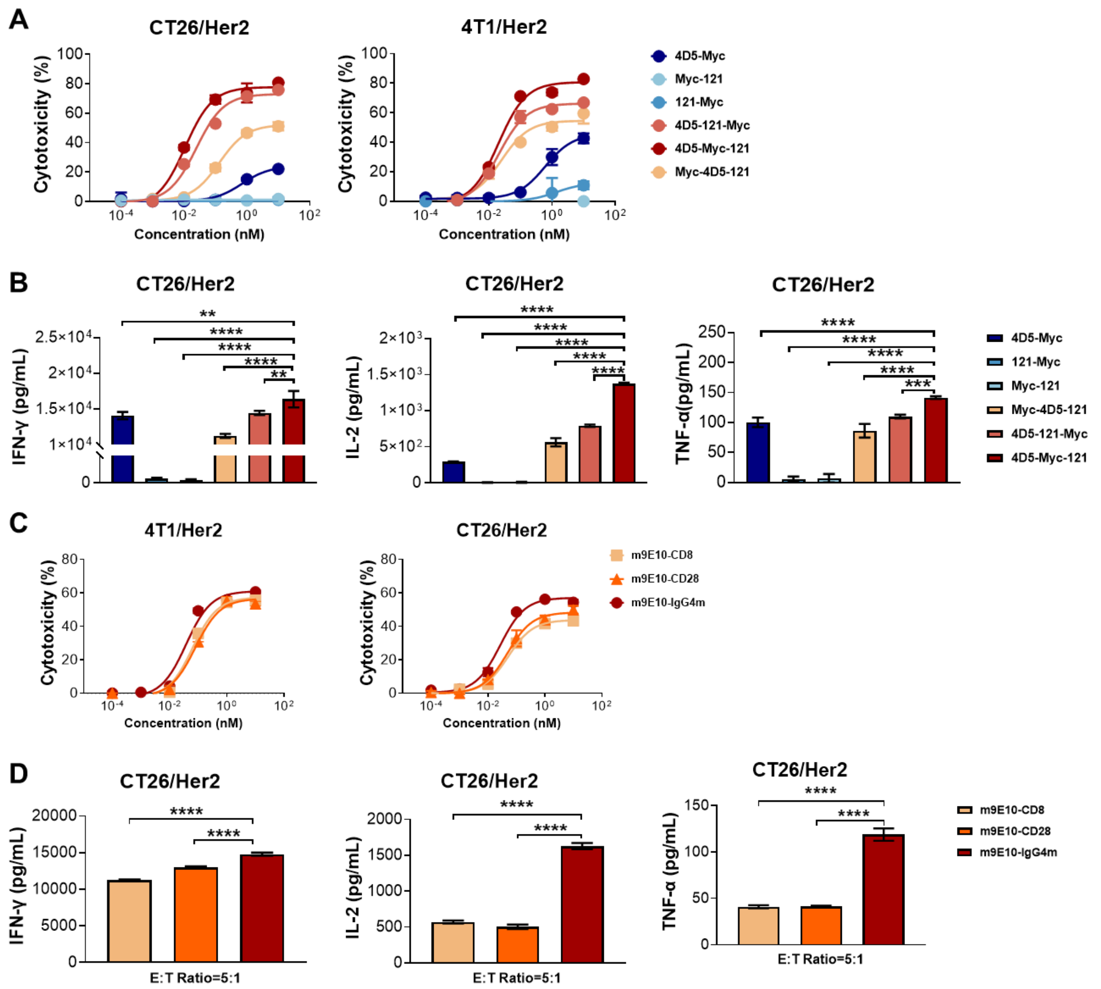


**Figure S14. Achieving optimal antitumor efficacy by optimizing the switch structure and hinge domain on murine 9E10-CAR-T cells. Related to Figure 5.**

(A) Cytotoxicity of m9E10-IgG4m CAR-T cells with different switches to the CT26/Her2 and 4T1/Her2 cell lines. The mCAR-T cells and target cells were coincubated with different switches ($10^{-4}$~$10^{1}$ nM) at an E:T ratio of 5:1 for 24 h. Cytolytic activity was evaluated via an LDH release assay (n=3). (B) Mouse IL-2, IFN-γ and TNF-α detection. m9E10-IgG4m CAR-T cells with different switches to the CT26/Her2 cell line. CAR-T cells and target cells were coincubated with different switches (1 nM) at an E:T ratio of 5:1 for 24 h. Cytokine levels were determined via ELISA (n=3). **$P<0.01$, ***$P<0.001$ and ****$P<0.0001$ by one-way ANOVA, means ± SDs. (C) Cytotoxicity of murine universal receptor CAR-T cells with different hinges to the CT26/Her2 and 4T1/Her2 cell lines. CAR-T cells and target cells were coincubated with different concentrations of 4D5-Myc-121 at an E:T ratio of 5:1 for 24 h. Cytolytic activity was evaluated via an LDH release assay (n=3). (D) Mouse IL-2, IFN-γ and TNF-α detection. m9E10-CD8, m9E10-CD28 and m9E10-IgG4m CAR-T cells were coincubated with CT26/Her2 cells and 1 nM 4D5-Myc-121 at an E:T ratio of 5:1 for 24 h. Cytokine levels were determined via ELISA (n=3). ****$P<0.0001$ by one-way ANOVA, means ± SDs.

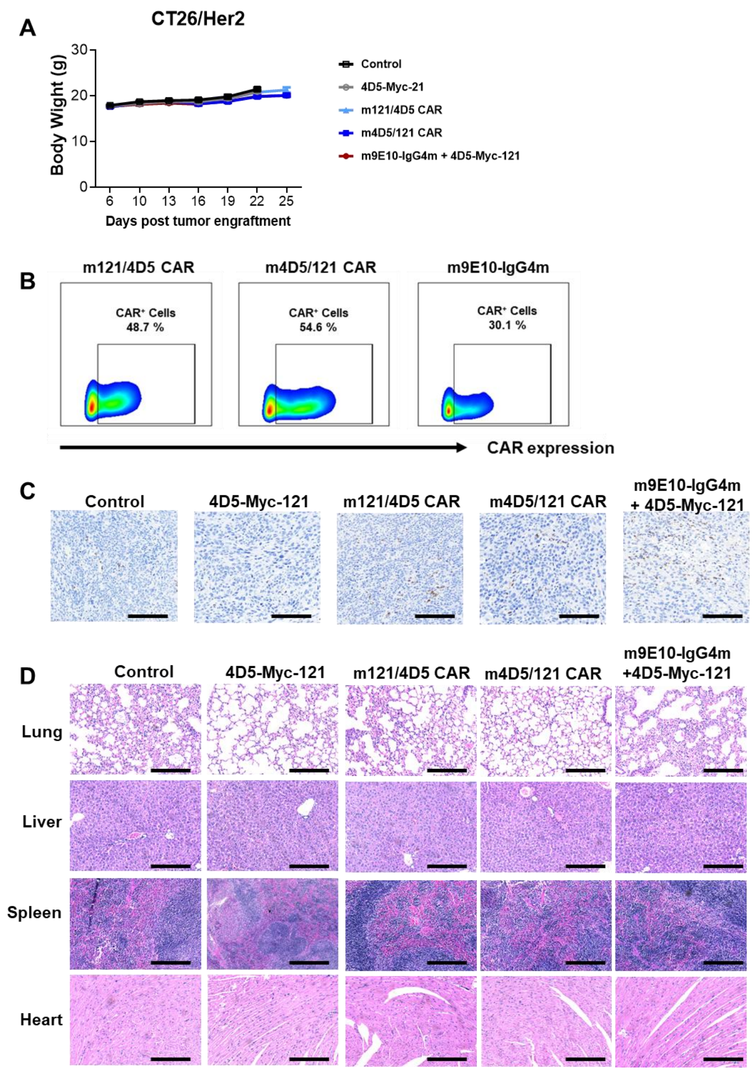


**Figure S15. Validation of sCAR-T using syngeneic tumor models. Related to Figure 5.**

(A) Mouse weight changes of the CT26/Her2 tumor model from the day after CAR-T cells injection (n=5). (B) m9E10-CAR-T cells were detected by flow cytometry, and an anti-$(G4S)_n$ antibody was used to detect the G4S linker in the m4D5/121 CARs, m121/4D5 CARs and m9E10-IgG4m CARs. (C) Immunohistochemical staining of tumor-infiltrating T cells. The tumor tissue samples were labeled with a rabbit anti-mouse CD3 antibody, and then an HRP-conjugated rabbit monoclonal antibody was used as a secondary antibody (n=3). Scale bar, 100 μm. (D) Hematoxylin and eosin-stained lung, liver, spleen and heart samples from BALB/c mice in the CT26/Her2 tumor model (n=3) on day 14. Scale bar, 100 μm.

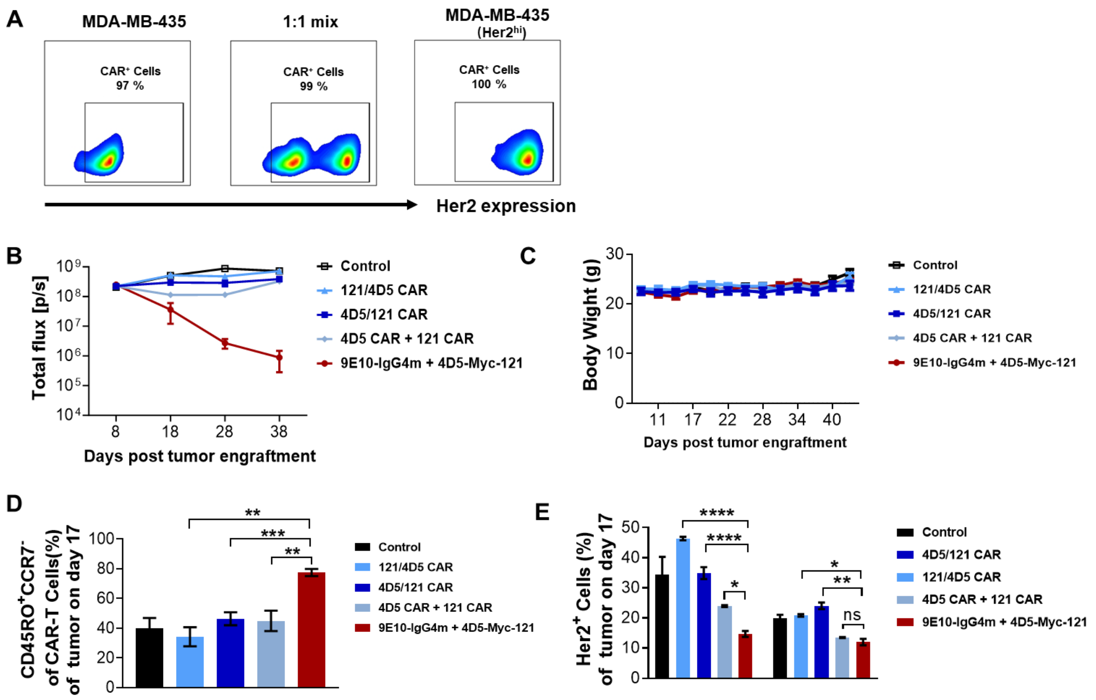


**Figure S16.** ***In vivo*** **validation of sCAR-T against immune escape. Related to Figure 6.**

(A) Her2 levels in the MDA-MB-435 and MDA-MB-435/Her2$^{hi}$ cell lines were detected via flow cytometry, and an anti-Her2 antibody was used. (B) Total flux [p/s] measurements of the mice (n=5). (C) Mouse weight changes in each group beginning on the day after CAR-T-cell injection (n=5). (D) Proportion of tumor-infiltrating CD45RO$^{+}$CCR7$^{-}$ effector memory subtype of CAR-T cells (n=4). **P < 0.01, ***P < 0.001 by one-way ANOVA, means ± SEM. (E) Her2 level in the tumor sample on day 17 (n=4). *P < 0.05, **P < 0.01, ****P < 0.0001 and ns=P>0.05 by two-way ANOVA, means ± SEM.

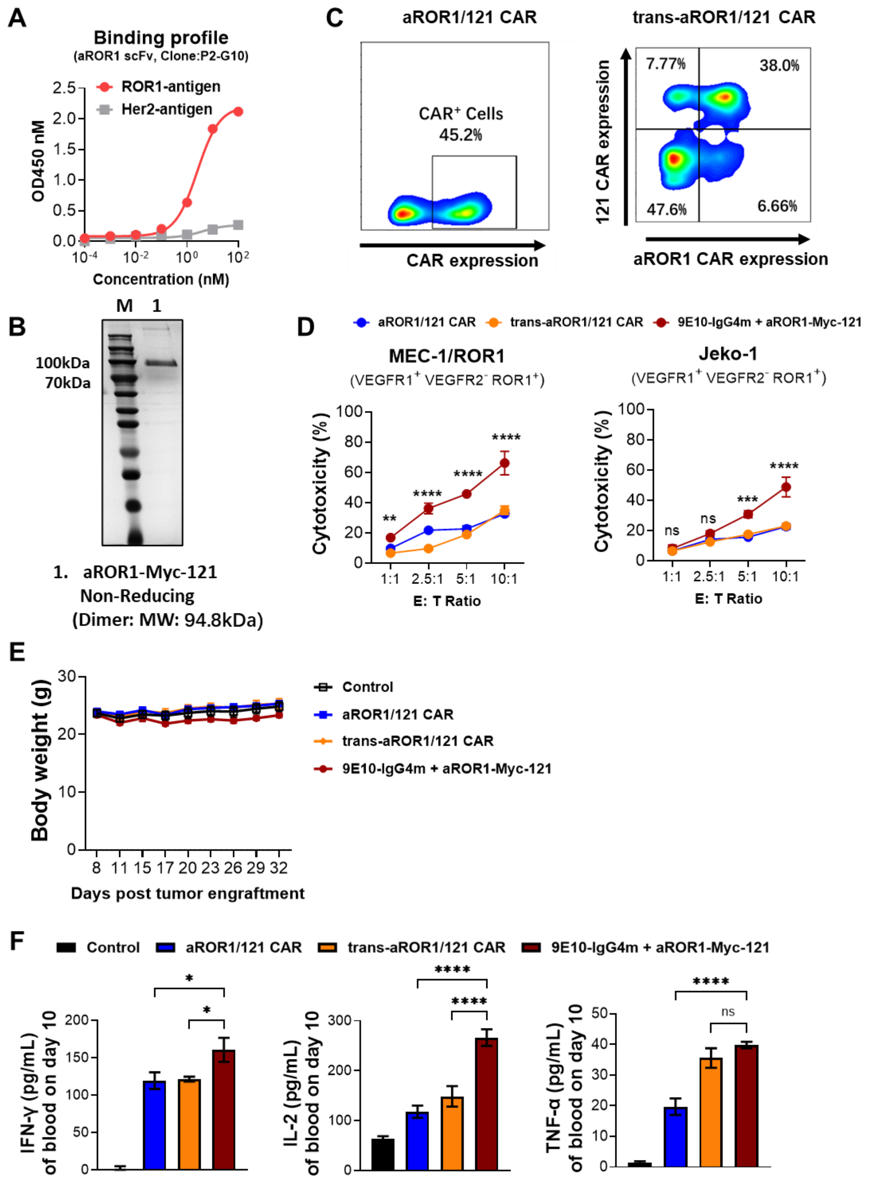


**Figure S17.** ***In vitro*** **and** ***in vivo*** **validation of anti-ROR1 sCAR-T cells. Related to Figure 7.** (A) Binding profiles of anti-ROR1 scFv (Clone: P2-G10) to the ROR1 antigen. (B) SDS–PAGE analysis of aROR1-Myc-121 under non-reducing conditions. (C) Flow cytometry detection of aROR1/121 CAR and trans-aROR1/121 CAR-T cells. An anti-mIgG (H+L) antibody was used to detect aROR1 in both CAR types, and an anti-FLAG antibody was used to detect VEGF121 in trans-aROR1/121 CARs. (D) Cytotoxicity of aROR1/121 CAR-T, trans-aROR1/121 CAR-T, and 9E10-IgG4m CAR-T cells in the presence of 1 nM aROR1-Myc-121 against multiple tumor cell lines at various E:T ratios for 24 h. Cytolytic activity was assessed by LDH release assay (n=3). Significance is marked as trans-4D5/121 CAR vs. 9E10-IgG4m + aROR1-Myc-121. **P < 0.01, ***P < 0.001 ****P < 0.0001 and ns=P>0.05 by two-way ANOVA, means ± SDs. (E) Body weight changes in each group starting from the day after CAR-T cell injection (n=5-7). (F) Serum levels of human IFN-γ, IL-2, and TNF-α on day 10 (n=5-7). *P < 0.05, ****P < 0.0001 and ns=P>0.05 by two-way ANOVA, means ± SEM.

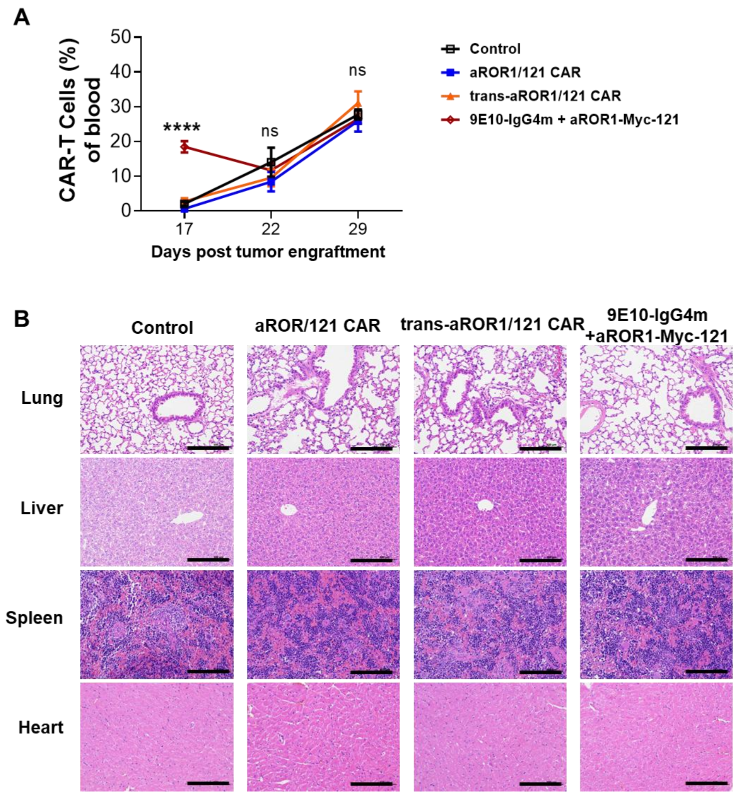


**Figure 18.** ***In vivo*** **analysis of the MDA-MB-468 tumor model. Related to Figure 7.**

(A) Proportion of circulating CAR-T cells in peripheral blood (n=5). Significance is marked as trans-aROR1/121 CAR vs. 9E10-IgG4m + aROR1-Myc-121, ***P < 0.001 and ns=P>0.05 by two-way ANOVA, means ± SEM. (B) Hematoxylin and eosin–stained sections of lung, liver, spleen, and heart from MDA-MB-468 tumor model (n=2) on day 14. Scale bar, 100 μm.

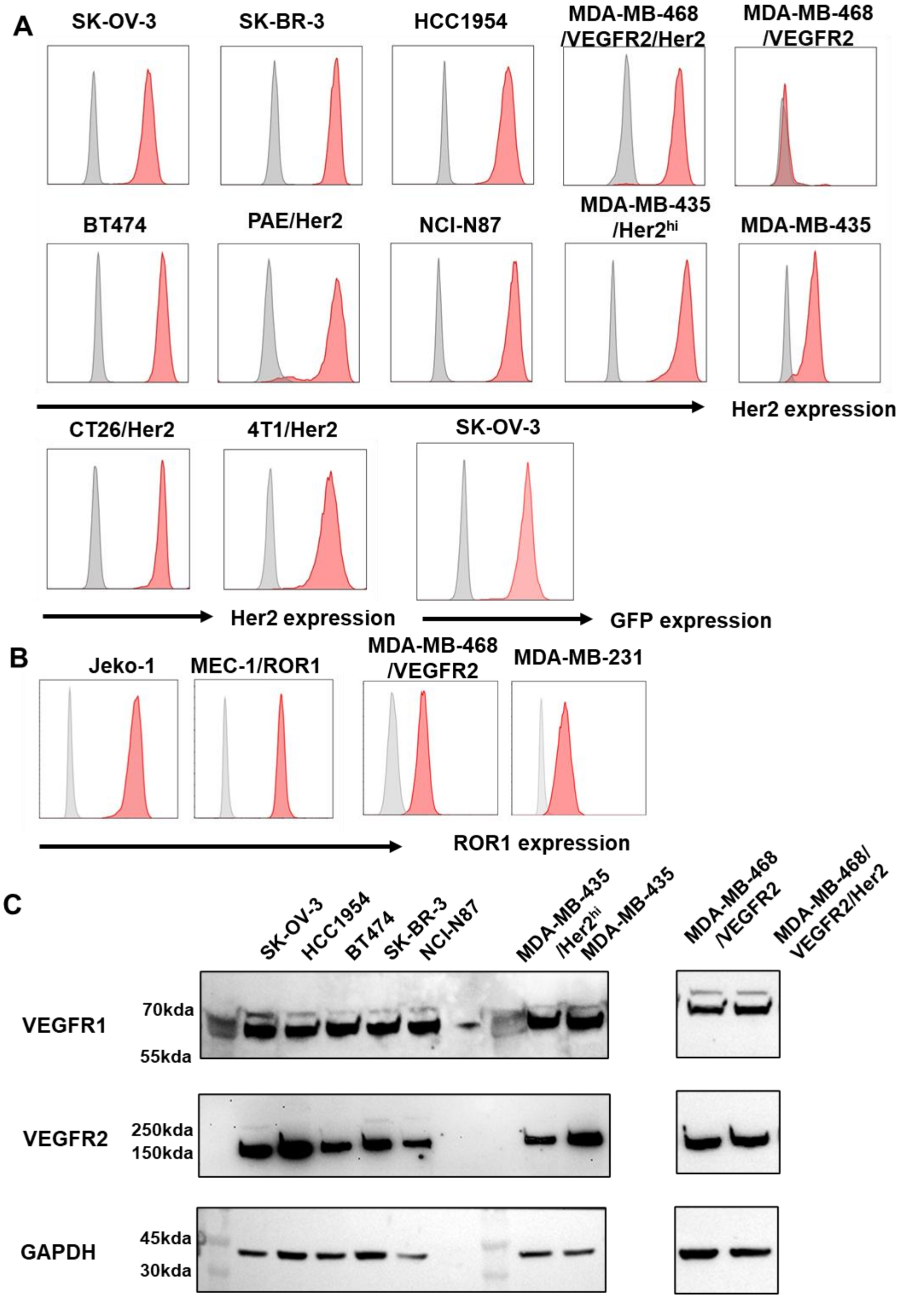


**Figure S19. Her2, ROR1, VEGFR1 and VEGFR2 detection in cell lines. Related to Figure 1, 2, 3, 4, 5, 6 and 7.**

(A) The surface expression of the Her2 antigen was evaluated using flow cytometry with an APC-conjugated anti-Her2 antibody. (B) Surface expression of ROR1 was assessed using an Alexa Fluor 647–conjugated anti-ROR1 antibody. (C) The VEGFR1 antigen was evaluated using a rabbit anti-human VEGFR1 antibody as the primary antibody and HRP-conjugated goat anti-rabbit IgG as the secondary antibody. The VEGFR2 antigen was evaluated using a rabbit anti-human VEGFR2 antibody as the primary antibody and HRP-conjugated goat anti-rabbit IgG as the secondary antibody.

**Table S1 Molecular weight identification via primary mass spectrometry. Related to Figure 2.**

## Mass spectrometric analysis

| Constructs | Non-Reducing Condition Expected/Observed Mass | Reducing Condition Expected/Observed Mass |
|---|---|---|
| **4D5-Myc** | 28794 Da / 28789 Da | 28718，94 Da / 28788 Da |
| **121-Myc** | 34330 Da / 34310 Da | 17165 Da / 17160 Da |
| **Myc-121** | 34330 Da /34312 Da | 17165 Da / 17157 Da |
| **4D5-121-Myc** | 87444 Da / 87474 Da | 43722 Da / 43713 Da |
| **4D5-Myc-121** | 88704 Da / 88680 Da | 44352 Da / 44346 Da |
| **Myc-4D5-121** | 86296 Da / 86298 Da | 43148 Da / 43139 Da |

Reduced samples were prepared by incubating with 1 mM DTT on ice for 1 h. The concentration of the nonreduced and reduced switch proteins was 1 mg/mL.

**Table S2 Oligomeric nature state identification via primary mass spectrometry. Related to Figure 2.**

## SEC-HPLC analysis

| Constructs | Retention (min) | % of total peak area | Asymmetry |
|---|---|---|---|
| **4D5-Myc** | 22.77 | 99.1 | 1.67 |
| **121-Myc** | 20.075 | 99.16 | 2.64 |
| **Myc-121** | 20.077 | 97.15 | 2.99 |
| **4D5-121-Myc** | 18.028 | 99.11 | 1.23 |
| **4D5-Myc-121** | 17.922 | 96.42 | 1.19 |
| **Myc-4D5-121** | 18.815 | 94.24 | 1.65 |

All switch proteins were purified by SEC-HPLC and concentrated to 0.5–1 mg/mL in PBS.
